# The iMab antibody selectively binds to intramolecular and intermolecular i-motif structures

**DOI:** 10.1093/nar/gkae1305

**Published:** 2025-01-15

**Authors:** Emanuela Ruggiero, Maja Marušič, Irene Zanin, Cristian David Peña Martinez, Daniel Christ, Janez Plavec, Sara N Richter

**Affiliations:** Department of Molecular Medicine, University of Padua, via A. Gabelli 63, 35121 Padua, Italy; Slovenian NMR Centre, National Institute of Chemistry, Hajdrihova 19, SI-1000 Ljubljana, Slovenia; Department of Molecular Medicine, University of Padua, via A. Gabelli 63, 35121 Padua, Italy; Garvan Institute of Medical Research, Immunology Department, 384 Victoria Street, Darlinghurst, Sydney, NSW 2010, Australia; St Vincent’s Clinical School, Faculty of Medicine, University of New South Wales, 384 Victoria Street, Darlinghurst, Sydney, NSW 2010, Australia; Garvan Institute of Medical Research, Immunology Department, 384 Victoria Street, Darlinghurst, Sydney, NSW 2010, Australia; St Vincent’s Clinical School, Faculty of Medicine, University of New South Wales, 384 Victoria Street, Darlinghurst, Sydney, NSW 2010, Australia; Slovenian NMR Centre, National Institute of Chemistry, Hajdrihova 19, SI-1000 Ljubljana, Slovenia; Department of Molecular Medicine, University of Padua, via A. Gabelli 63, 35121 Padua, Italy; Microbiology and Virology Unit, Padua University Hospital, via Giustiniani 2, 35128 Padua, Italy

## Abstract

i-Motifs (iMs) are quadruplex nucleic acid conformations that form in cytosine-rich regions. Because of their acidic pH dependence, iMs were thought to form only *in vitro*. The recent development of an iM-selective antibody, iMab, has allowed iM detection in cells, which revealed their presence at gene promoters and their cell cycle dependence. However, recent evidence emerged which appeared to suggest that iMab recognizes C-rich sequences regardless of their iM conformation. To further investigate the selectivity of iMab, we examined the binding of iMab to C-rich sequences, using a combination of pull-down and western blot assays. Here, we observe that the composition of buffers used during binding and washing steps strongly influences the selectivity of antibody binding. In addition, we demonstrate by nuclear magnetic resonance that several of the previously reported C-rich sequences, which were not expected to form iMs, actually form intermolecular iMs which are selectively recognized by iMab. Our results highlight the specificity of the iMab antibody, emphasize the importance of avoiding *in vitro* artifacts by optimizing DNA concentrations, blocking and washing conditions, and confirm that iMab is selective not only for intramolecular iMs but also for intermolecular iMs, while not affecting the iM conformation.

## Graphical Abstract

**Graphical Abstract:**
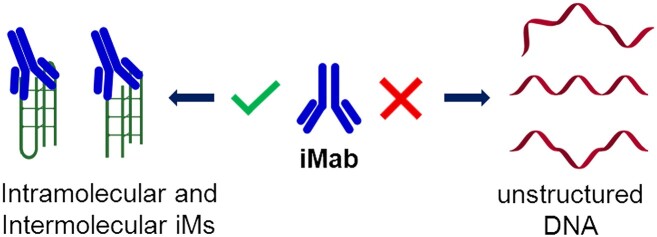

## Introduction

The high dynamics of DNA during cellular processes allows it to adopt several conformations alternative to the double helix, including quadruplexes such as i-motifs (iMs) and G-quadruplexes (G4s). iMs form within cytosine (C)-rich regions through the intercalation of hemi-protonated C^+^-C base pairs, when a minimum of four tracts of at least two consecutive Cs are present (1). G4s occur within guanine (G)-rich regions when two or more G-tetrads, which are associations of four Gs, self-stack (2). While G4s have been extensively studied thanks to the availability of specific antibodies (3) and ligands (4,5) that recognize and bind them, iMs have been much less explored. Prediction algorithms have located putative iM-forming sequences in key regulatory regions, such as gene promoters, centromeres and telomeres (6). Many studies have investigated iM formation experimentally *in vitro*, contributing to the understanding of its folding factors (6). The development of the first anti-iM antibody, iMab (7), was a breakthrough in iM research, leading to the detection and mapping of iMs in cells (8). iMab has been successfully used in several *in vitro* techniques, such as dot blot (9,10) and pull-down (11) assays, and has been used to develop a custom microarray for the screening of thousands of iM-forming sequences (12), which has expanded iM structural characterization. In cells, immunofluorescence with iMab localized iMs in the nucleus of several human cell lines (7) and showed that their number was cell cycle dependent. iMs were reported to be most abundant during active transcription phases, such as G1, and to decrease in subsequent phases (13), implicating iMs in cell regulatory roles. Recently, iMab has been used in different high-throughput sequencing-based techniques, iMab-IP-Seq (9,14) and CUT&Tag (11), providing precise information on the distribution and location of iMs in the whole genome. In the case of iMab-IP-Seq, an initial analysis was performed on purified DNA extracted from the rice plant, where iMs showed an intrinsic subgenomic distribution and *cis*-regulatory function (9); methylation of the immunoprecipitated region was found to strongly influence iM formation, revealing new aspects in genome regulation (10). When the analysis was performed on purified human genomic DNA, it demonstrated the wide distribution of sequences capable of iM formation, which are common among highly expressed genes and those upregulated in G0/G1 cell cycle phase (14). In the context of chromatin, our group recently applied iMab to the CUT&Tag protocol on two different human cells. We found that iMs in cells are mainly located at actively transcribing gene promoters in open chromatin regions, and that their abundance and distribution are specific to each cell type. iMs with both long and short C-tracts were recovered, and their folding was further confirmed *in vitro* (11). The data obtained with the iMab-CUT&Tag were then used to develop a machine learning pipeline called iM-Seeker, which aims to predict both the folding status and the structural stability of iMs, based on experimental evidence (15).

In contrast to the above studies, a recent nuclear magnetic resonance (NMR) study indicated that synthetic iM-forming oligonucleotides inserted into the nucleus are often not folded, suggesting that the nuclear environment may not be broadly supportive of iM folding (16). Recently, a study by Boissieras *et al.* suggested that the iMab antibody is capable of binding to C-rich synthetic DNA oligonucleotides independent of iM formation, and that it stimulates iM unfolding (17). These apparent discrepancies are not unexpected due to the transient nature of iM formation, and the use of different experimental methods and conditions, including buffer conditions and DNA organization (purified genomic DNA *vs* chromatin *vs* synthetic oligonucleotides) (18). To address these issues, we have investigated the selectivity of iMab towards C-rich sequences using a pull-down/western blot (WB) approach (11). We report the importance of optimizing condition of each key step, highlight the specificity of iMab, and demonstrate that this antibody selectively recognizes both intramolecular and intermolecular iMs, while at the same time not affecting iM conformation.

## Materials and methods

### Oligonucleotides

Oligonucleotides used in the CD and pull-down assays were purchased from Sigma-Aldrich and are listed in Table 1. For the pull-down assay, a biotin-TEG residue was added at the oligonucleotide 3′-end.

**Table 1.** Oligonucleotides used in this study. C-tracts composed of two or more Cs are shown in bold

Name	Sequence (5′-3′)
LTR-IIIc	**CCCC**AGT**CCC**G**CCC**AGG**CC**ACG**CC**T**CCC**
Random	CAATCTCAATCTCAATCTCAATCT
hTeloC	TAA**CCC**TAA**CCC**TAA**CCC**TAA**CCC**TAA
hTeloC 3×2	TAATAA**CC**TAA**CC**TAA**CC**TAATAATAA
hTeloC 3×3	TAATAATAA**CCC**TAA**CCC**TAA**CCC**TAA
hTeloC 3×4	TAATAA**CCCC**TAA**CCCC**TAA**CCCC**TAA
hTeloC 4×2	TAA**CC**TAA**CC**TAA**CC**TAA**CC**TAA
hTeloC 4×4	TAA**CCCC**TAA**CCCC**TAA**CCCC**TAA**CCCC**TAA
hTeloCnf (no flanking)	**CCC**TAA**CCC**TAA**CCC**TAA**CCC**
hTeloC 3×2nf (no flanking)	**CC**TAA**CC**TAA**CC**
hTeloC 3×3nf (no flanking)	**CCC**TAA**CCC**TAA**CCC**
hTeloC 3×4nf (no flanking)	**CCCC**TAA**CCCC**TAA**CCCC**
hTeloC 3×3 1f (1-nt flanking)	A**CCC**TAA**CCC**TAA**CCC**T
hTeloC 3×3 3f (3-nt flanking)	TAA**CCC**TAA**CCC**TAA**CCC**TAA
hTeloC scra	TACACTCACACTCACACTCACACTCAA

### Circular dichroism

Oligonucleotides used for circular dichroism (CD) analysis were diluted to 3 μM in 20 mM phosphate buffer, 80 mM KCl at pH 5.4, 6.0 and 7.4. Samples were heated at 95°C for 5 min and then slowly cooled to room temperature overnight. 5 mm and 1 mm optical-length quartz cells were used to record CD spectra on a Chirascan-Plus equipped with a Peltier temperature controller. CD spectra were performed at 20°C and over a temperature range of 20–90°C, and data were acquired from wavelength 230 to 320 nm. In the presence of iMab, oligonucleotides were folded as previously described, binding was performed for 5 min on ice, and the CD spectrum was recorded immediately. CD data were baseline-corrected and the observed ellipticities were converted to mean residue ellipticity according to $\theta$ = degree × cm^2^ × dmol^-1^ (molar ellipticity). CD analysis was performed in duplicate and values were plotted using R as the mean of the two independent replicates (19).

### iMab pull-down and western blot

Pull-down coupled with WB was adapted from previously described procedures (11). In brief, samples were prepared by diluting biotinylated oligonucleotides (Table 1) to 1.5 or 0.3 μM in 100 mM phosphate buffer pH 6.0, denaturing at 95°C for 5 min and cooling overnight. Upon immobilization of the samples on streptavidin-coated magnetic beads (Dynabeads™ M-280 Streptavidin, ThermoFisher Scientific, #11205D), oligonucleotides were incubated with 100 or 10 ng FLAG-tagged iMab (expressed as previously described (20) or alternatively provided by Absolute antibody, #Ab01462-30.135) for the indicated time in ice bath in the appropriate binding buffer (Tris–HCl pH 7.5 10 mM, MgCl_2_ 1 mM, KCl 10 mM, dithiothreitol (DTT) 1 mM), enriched with blocking agents and NaCl as described in Figure 2. Samples were washed four times in wash buffer (50 mM Tris–HCl pH 7.5/NaCl at different concentrations) and once with Phosphate-buffered saline (PBS). WB was performed according to known procedures (21). Samples were loaded on a 10% SDS-PAGE and the gel was then transferred on a Polyvinylidene fluoride (PVDF) membrane. The latter was blocked in 2.5% PBS-milk buffer for 1 h, incubated with the anti-FLAG antibody 1:1000 (Sigma-Aldrich, #F3165), washed in 0.1% PBS-tween, and finally incubated with secondary goat anti-mouse 1:5000 horseradish peroxidase (HRP) antibody (Merck-Millipore #12–349). Images were acquired on the Alliance Uvitec (Uvitec Ltd. Cambridge, Cambridge, United Kingdom) instrument by HRP bioluminescence measurement.

### Nuclear magnetic resonance

DNA oligonucleotides were synthesized with DNA/RNA H-8 K&A Laborgereate GbR synthesizer using standard solid-phase phosphoramidite chemistry in a DMT-ON mode, deprotected with AMA (Ammonium Hydroxide/40% aqueous MethylAmine 1:1 v/v), purified with Glen-Pak™ cartridges and desalted on ÄKTA Purifier with HiPrep 26/10 Desalting column. Samples were prepared at 0.1 or 1 mM oligonucleotide concentration in buffer (20 mM potassium phosphate buffer, pH 5.4, 6.0 or 7.4, and 80 mM KCl) in the absence or presence of MgCl_2_ (1 mM MgCl_2_, 10 mM KCl, 100 mM potassium phosphate buffer at pH 5.4 or 6.0) and 10% D_2_O. Samples were annealed by heating to 95°C and cooling to room temperature overnight. 1D ^1^H NMR spectra were recorded on Bruker AVANCE Neo 600 MHz NMR spectrometer with 256 scans, spectral width of 24.5244 ppm and interscan delay of 2 s. Excitation sculpting was used for the suppression of water signal. Spectra were processed, analyzed and integrated with TopSpin 4.1.4 (Bruker). NMR melting experiments were conducted at 5°C intervals, with waiting periods of either 10 or 20 min to allow for temperature equilibration. After the waiting period, the NMR spectrometer was prepared for recording, which required an additional 20 min. Therefore, the total time spent by the sample at a given temperature before data acquisition was either 30 or 40 min, and the total time for a single point of NMR melt experiment was 50 or 60 min.

## Results

### Optimization of iMab binding conditions

In order to expand our knowledge of iM structures formed by C-rich sequences, we set up a combined pull-down/western blot (WB) experimental approach to assess iMab specificity. We selected seven sequences (Table 1) as follows: two previously characterized iMs, namely the LTR-IIIc sequence located within the HIV-1 virus promoter (22) and the hTeloC sequence from the C-rich region of the human telomere (23), both reported to fold into stable iMs, as positive control sequences; a Random sequence unable to fold into stable DNA secondary structures as negative control. In addition, following Boisseras *et al.* (17), we included three other sequences derived from the hTeloC, all containing three C-tracts of two (hTeloC 3×2), three (hTeloC 3×3) or four (hTeloC 3×4) Cs each, flanked by TAA tracts to reach all the same length. Finally, an additional negative control (hTeloC scra), derived from the reshuffling of the hTeloC primary sequence, was included.

**Figure 1.**
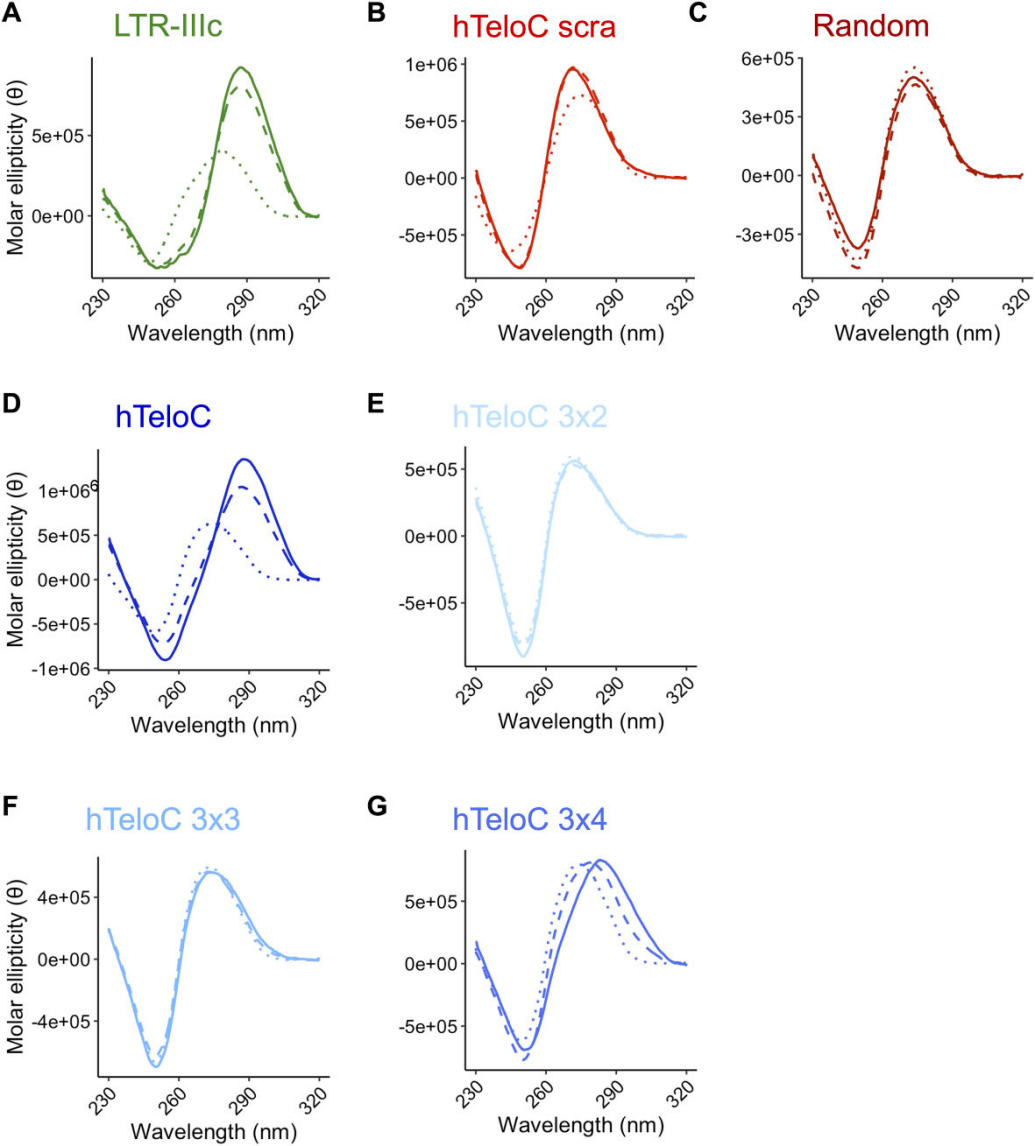
CD spectra of the biotinylated sequences used in the pull-down/WB assay. Samples were prepared in 20 mM phosphate buffer at pH 5.4 (plain line), pH 6.0 (dashed line) and pH 7.4 (dotted line) with 80 mM KCl, at 3 μM final concentration. Molar ellipticity (θ) was measured at 20°C and reported as θ = deg × cm^2^ × dmol^-1^.

First, we defined the folding profile of the selected biotinylated sequences to be used in the subsequent pull-down/WB analysis by CD performed at 20°C at increasing pH from 5.4 to 7.4 (Figure 1). We observed that the positive controls LTR-IIIc (Figure 1A) and hTeloC (Figure 1D) were iM folded in a pH-dependent manner, showing a positive peak around λ = 285 nm and a negative one around λ = 260 nm, as expected (24). The hTeloC scra and Random sequence (Figure 1B and C) displayed a CD spectrum characteristic of unfolded DNA (25). For the hTeloC variants, hTeloC 3×4 was found to be folded at acidic pH (Figure 1G), while the hTeloC 3×3 and hTeloC 3×2 showed an unfolded CD signature (Figure 1F and E). We also evaluated the CD spectra of the same unbiotinylated sequences and observed no major changes (Supplementary Figure S1), indicating that the presence of the terminal biotin residue does not significantly affect their three-dimensional fold. We then performed CD-melting experiments to assess the folding stability of representative sequences, namely hTeloC, hTeloC 3×3, hTeloC scra. The analysis was carried out at pH 5.4, 6.0 and 7.4, in the absence and presence of the biotin residue (Supplementary Figures S2–S4). As expected, the melting temperature was measurable only for the hTeloC sequence in iM-forming conditions at acidic pH: the *T*_m_ values calculated at pH 5.4 and 6.0 were around 38°C and 35°C, respectively, and were not affected by the presence of biotin (Supplementary Figure S4A). The melting profile did not show formation of folding intermediates. For hTeloC 3×3 and scra sequences, a proper temperature-dependent transition from a folded to an unfolded state was not observed, confirming that, in the tested conditions, both hTeloC 3×3 and scra sequences do not adopt alternative secondary structures.

**Figure 2.**
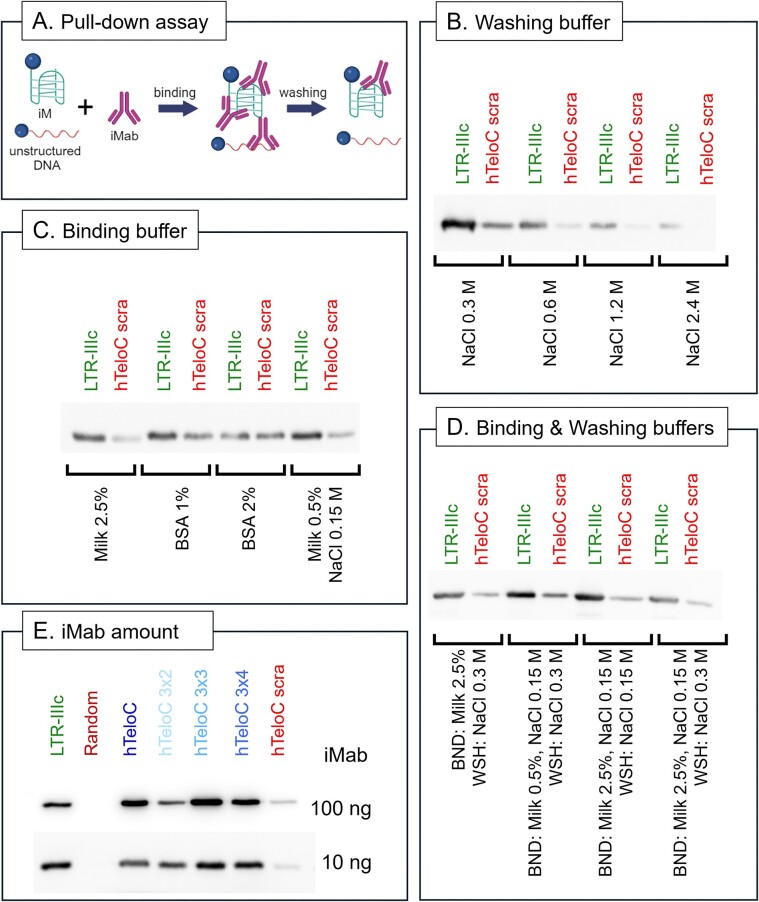
Assessment of iMab selectivity by pull-down/WB assay. (**A**) Cartoon model of the pull-down experimental approach. The biotinylated oligonucleotides were immobilized on streptavidin beads and incubated with iMab in the binding buffer indicated in panels (C) and (D). Subsequently, the obtained complexes were subjected to multiple wash steps in the buffer indicated in panels (A) and (D) to remove weak and unspecific interactions. (**B**) Evaluation of the effect of increasing ionic strength in the washing buffer, from 0.3 to 2.4 M NaCl. (**C**) Evaluation of the effect of blocking agents (skim milk or BSA at different concentrations) and ionic strength (NaCl) in the binding buffer. (**D**) Binding (BND) and washing (WSH) buffer optimization. (**E**) Pull-down followed by WB analysis on all sequences of interest at two different iMab concentrations, performed in the optimized conditions: binding buffer (milk 2.5%, NaCl 0.15 M), washing buffer (NaCl 0.15 M).

Following the determination of the folding state of our target sequences, pull-down assays were performed. In this protocol, the folded biotinylated oligonucleotides are first immobilized onto streptavidin-coated magnetic beads and incubated with the protein of interest (in our case the iMab antibody). Beads are then washed several times to remove non-specific and weak interactions (Figure 2A); the retained bound antibody is visualized by WB. We initially selected a known positive iM (LTR-IIIc) (22) and a negative sequence (hTeloC scra) and started optimizing both washing and binding conditions to obtain the best selectivity settings for iMab binding. First, we maximized the efficiency of the washing buffer by testing the ionic strength, using NaCl concentrations from 0.3 to 2.4 M (Figure 2B). We observed that, in general, LTR-IIIc showed a more intense band in the WB compared to the hTeloC scra, and, for both sequences, the binding became weaker with increasing NaCl concentrations. Therefore, in the following steps, we used concentrations of 0.15 or 0.3 M NaCl during the washing phase. As for the binding buffer, we evaluated the effect of the presence of different blocking agents, such as skim milk and Bovine Serum Albumin (BSA) protein, at different concentrations as shown in Figure 2C. We also included in the analysis a sample containing additional NaCl to increase the ionic strength during the binding step. We confirmed that iMab preferentially binds to the positive iM oligonucleotide, LTR-IIIc, and to a much lesser extent to the hTeloC scra. We observed that skim milk was more efficient than BSA in improving the selectivity for the iM folded sequence. The addition of ionic strength also improved selectivity. Therefore, we combined the results obtained in the first two settings and tested (i) the concentration of skim milk (0.5% or 2.5%) to which 0.15 M NaCl was added; (ii) the ionic strength of the washing buffer (0.15 or 0.3 M) (Figure 2D). Overall, the most selective iMab binding was obtained with 2.5% skim milk and 0.15 M NaCl in the binding buffer and 0.15 M NaCl in the washing buffer. Based on these data, we applied the optimized protocol to all the selected sequences (Figure 2E, top panel) and observed that iMab was able to bind to all iM-forming oligonucleotides (LTR-IIIc, hTeloC and hTelo C 3×4), while the Random fragment showed no band. A clear band was also observed for the hTeloC 3×2 and 3×3 sequences, and a weaker band for the negative control hTeloC scra. We next evaluated the amount of antibody used in the analysis, by reducing iMab concentration by 10-fold (Figure 2E, bottom panel): we observed no reduction in band intensity in any of the tested sequences, except for hTeloC scra, for which binding largely disappeared.

### iMab binds to both intramolecular and intermolecular iMs

**Figure 3.**
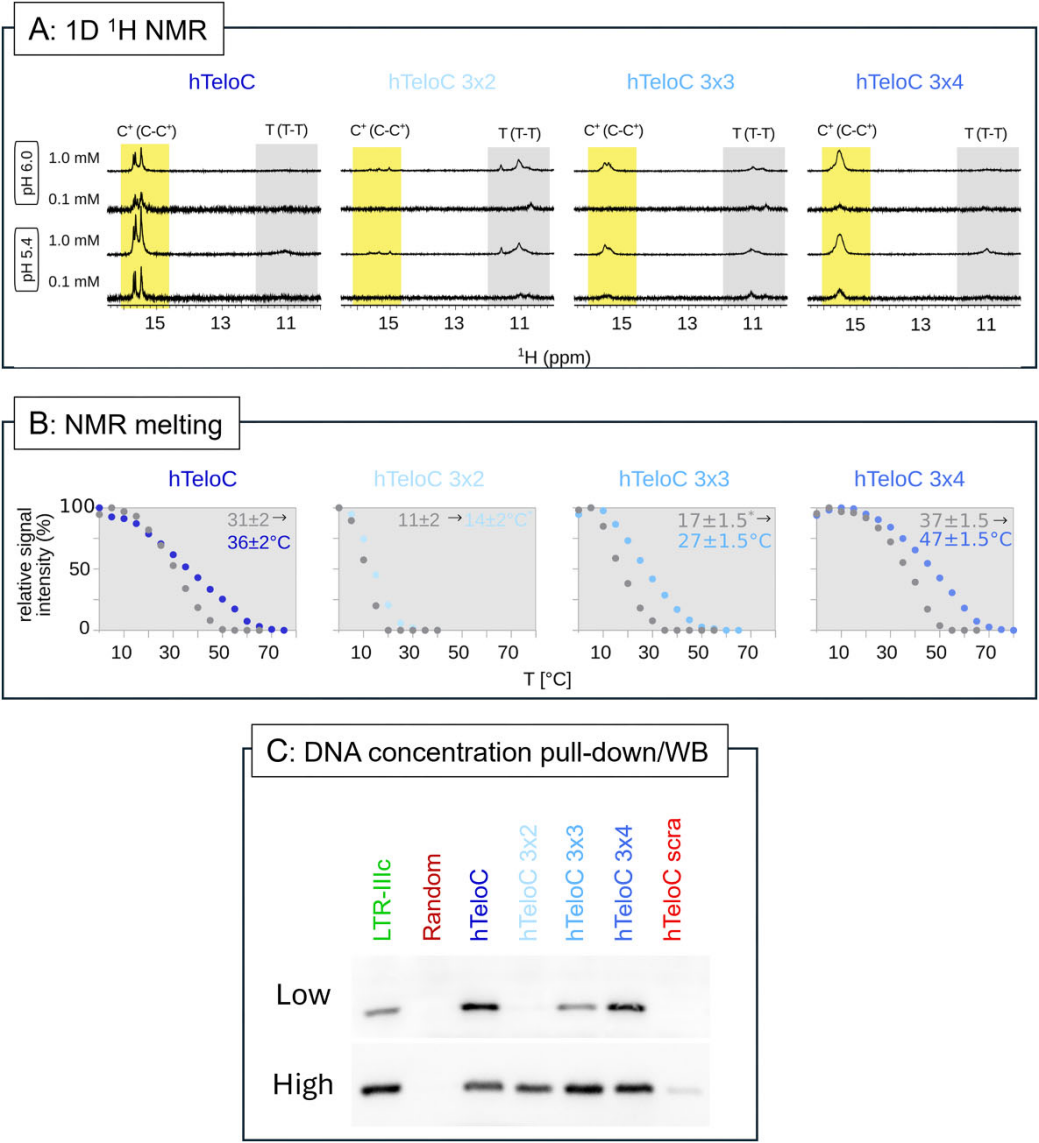
iMab binding profile to intermolecular iMs. (**A**) Imino region of 1D ^1^H NMR spectra recorded at 5°C, at different pH and oligonucleotide concentrations. Regions characteristic for signals of protons included in non-canonical C-C^+^ and T-T base-pairs are highlighted by the yellow and gray areas, respectively. The vertical scale of the spectra at 1.0 mM oligonucleotide concentration is reduced by a factor of 10. (**B**) *T*_1/2(NMR)_ at two different oligonucleotide concentrations obtained from an average of NMR melting and annealing curves based on the intensity of the signals in the imino region. Data points in the temperature profiles displayed in gray represent data at 0.1 mM oligonucleotide concentration, while data points in shades of blue represent data at 1.0 mM oligonucleotide concentration. *T*_1/2(NMR)_ labeled with asterisks have higher than average associated error; for complete analysis of the NMR melting data together with 1D proton NMR spectra see Supplementary Figures S5 and S6. (**C**) Pull-down/WB performed at low (300 nM) and high (1500 nM) DNA amounts, with 10 ng iMab per sample.

Although we were able to identify the best conditions for iMab to recognize its target, we still observed a clear WB band for the hTeloC 3×2 and 3×3 sequences, which, based on their sequence and our initial CD analysis, could not form intramolecular iM structures. However, we reasoned that both oligonucleotides could form intermolecular iM structures (26), which would explain their binding to iMab. Multimolecular iMs have mainly been studied for their application in nanotechnology and it is generally reported that they form in sequences with short C-tracts (26,27), and therefore show lower stability than intramolecular structures (28). To test our hypothesis, we performed ^1^H NMR analysis at two different oligonucleotide concentrations (Figure 3A), since high DNA concentrations stimulate the formation of intermolecular structures (29). Signals between δ 15 and 16 ppm are characteristic of protonated C residues found in iMs (29) and were observed for hTeloC and hTeloC 3×4 oligonucleotides, regardless of DNA concentration and at both pH 5.4 and 6.0, indicating the formation of stable iMs. Interestingly, the hTeloC 3×2 and 3×3 sequences also displayed signals characteristic of protonated C residues, albeit predominantly at high DNA concentrations and with a greater dependence on pH, supporting the formation of intermolecular structures with low stability. To deepen our analysis, we examined the NMR melting profiles and their concentration-dependence in the entire imino region (Figure 3B) and in each specific type of base pair (Supplementary Figures S5 and S6).

Our data show a significant increase in thermal stability of hTeloC, hTeloC 3×4 and hTeloC 3×3 oligonucleotides (5–10°C) at high oligonucleotide concentration, but lower stabilization of hTeloC 3×2 (3°C), which exhibited the lowest stability among the iMs. These results, in conjunction with the broad signal shape observed in Figure 3B and Supplementary Figures S5 and S6, are consistent with the formation of intermolecular iMs (28). The NMR melting curves showed higher cooperativity at the lowest DNA concentration, as expected due to the reduced presence of intermolecular interactions (30). The NMR analysis was next repeated using the same buffer used for iMab binding, to assess the effect of MgCl_2_ and increased KCl concentration on the folding, but no significant difference in the types of the structures present in solution was observed (Supplementary Figures S7 and S8). We hence performed pull-down/WB analysis at lower DNA concentrations to minimize the formation of intermolecular iMs and compared it to the binding obtained at higher DNA concentration (Figure 3C). We found that at low DNA concentration iMab binding was robust for monomolecular iM-forming sequences, i.e. those sequences that showed NMR iM signature peaks at both high and low concentrations (LTR-IIIc, hTeloC, hTeloC 3×4), whereas it was strongly reduced (hTeloC 3×3) or completely lost (hTeloC 3×2) for those sequences that could only form intermolecular iMs, i.e. those sequences that showed NMR iM signature peaks only at high DNA concentrations. No band was observed in the negative controls. We additionally tested two hTeloC derivatives with different length of four cytosine stretches: iM hTeloC 4×4 and hTeloC 4×2. Their folding and stability were initially assessed by CD at different pH values: we observed pH-dependent iM formation of hTeloC 4×4, while hTeloC 4×2 did not fold into iM (Supplementary Figures S9 and S10), as already observed for hTeloC 3×2 and hTeloC 3×3 in these same conditions (Figure 1 and Supplementary Figures S1–S4). Again, no differences between the biotinylated and non-biotinylated oligonucleotides were observed (Supplementary Figures S9 and S10). Similarly to the data shown in Figure 3C, also in this case iMab binding was concentration-dependent for hTeloC 4×2, supporting formation of intermolecular iM (Supplementary Figure S11). In contrast iMab bound strongly to hTeloC 4×4, which forms a stable intramolecular iM (Supplementary Figure S11).

### Flanking regions affect the formation of intermolecular iMs

NMR profiles revealed a prominent presence of signals characteristic for T-T base-pairs (Figure 3A), especially in hTeloC 3×2 and hTeloC 3×3 sequences, potentially impacting the three-dimensional folding of the tested oligonucleotides. To investigate this further, we characterized hTeloC-derived sequences with the following modified flanking TAA regions: (i) complete removal of bases flanking the C-tracts, (ii) inclusion of a single nucleotide flanking the C-tracts and (iii) addition of a single TAA residue at both sides of the sequence (Table 1). We assessed the folding patterns of these modified versions using hTeloC 3×3 as a reference sequence and observed that, in the absence of flanking TAA tracts, iM folding was more distinct in both CD and NMR spectra (Supplementary Figure S12). Based on these findings, we proceeded with sequences devoid of flanking nucleotides for further analysis.

We extended the CD analysis to all hTeloC no flanking (nf)-derived sequences across three reference pH values: 5.4, 6.0 and 7.4 (Supplementary Figure S13). Notably, we observed a marked increase in the transitional pH of intramolecular iMs, as both hTeloCnf and hTeloC 3×4nf displayed very stable folding at pH 6.0 when compared to the sequences with the flanking nucleotides (Figure 1). For intermolecular iMs, the hTeloC 3×3nf sequence exhibited a distinct iM profile under acidic conditions, whereas the CD spectrum of hTeloC 3×2nf suggested the predominance of an unfolded component at the tested concentration. Since we have shown that intermolecular iMs form at high DNA concentrations, we conducted a concentration-dependent investigation and demonstrated that at higher oligonucleotide concentrations, hTeloC 3×3nf folded to iM at acidic pH (Supplementary Figure S14A). Regarding hTeloC 3×2nf, a shift towards higher wavelengths was evident at higher concentrations, suggesting the adoption of the intermolecular iM conformation (Supplementary Figure S14B), thus supporting the signal observed in WB analysis (Supplementary Figure S11). To complete our investigation, the folding of the sequences without flanking nucleotides was also examined by 1D proton NMR, evaluating the influence of pH, temperature and oligonucleotide concentration (Supplementary Figure S15). At pH 5.4 and low temperature (0°C), all hTeloCnf-derived sequences exhibited imino signals corresponding to C^+^-C base-pair formation, independent of DNA concentration. Shifting to room temperature maintained the folded state in all sequences except hTeloC 3×2nf, the least stable iM, consistent with CD results. At neutral pH, folding was primarily detectable at low temperature and 1 mM concentration for the stable sequences hTeloCnf and hTeloC 3×4nf, as well as for the hTeloC 3×3nf.

### iMab binds rapidly and specifically to intra- and intermolecular iMs

We next investigated whether the neutral pH of the binding buffer might disrupt iM folding, potentially leading to iMab binding to unfolded sequences, as previously suggested by Boissieras *et al.* (17). To assess this, we performed a time-resolved CD analysis on the iM-forming sequence hTeloC after adjusting the solution to neutral pH using K_2_HPO_4_. CD spectra were recorded every 2 min. Upon addition of potassium phosphate, iM folding was initially preserved, displaying a maximum peak at λ = 286.5 nm. A gradual redshift of the positive peak was observed over time, indicating progressive unfolding of the structure. Notably, the iM structure remained intact upon the first eight minutes after pH adjustment (Supplementary Figure S16A).

Based on these observations, we conducted the pull-down experiment with a reduced binding incubation time of five minutes to ensure the oligonucleotide remained in the folded state in solution. WB analysis (Supplementary Figure S16B) confirmed the same binding specificity observed after 1h incubation with iMab (Figure 3C), validating (i) iMab binding specificity for iM-forming sequences, (ii) iMab binding speed, consistently with previous reports (7), and (iii) the maintenance of iMab binding specificity also over extended exposure times.

Finally, we investigated the effect of iMab on the folding of selected oligonucleotides, i.e. hTeloCnf and hTeloC 3×3nf. CD analysis was conducted at pH 6.0, where hTeloCnf is stably folded, while hTeloC 3×3nf remains unfolded, and at pH 7.4 where both are unfolded in the CD conditions. This experimental setup enabled us to evaluate both the potential unfolding and folding effect of the antibody. Even at a 10-fold molar excess of iMab, no significant changes in the oligonucleotide conformation were detected, indicating that iMab binding neither disrupts nor induces the iM structure (Supplementary Figure S17).

In conclusion, our data confirm the selective and rapid binding of iMab to iM-forming sequences and demonstrate for the first time that the antibody recognizes both intramolecular and intermolecular iM structures without influencing their folding state.

## Discussion

The dynamic state of non-canonical nucleic acid structures has made their detection a challenge, especially within the environment of the cell. The development of the iMab antibody, the first to recognize iM DNA structures, has given a major boost to the research in this field. In fact, iMab has been used in several types of assays in the recent years: different laboratories have shown that iMs are folded in the genome of different species (9,11) and are localized in the nucleus of human cells (13). Recently, however, the selectivity of iMab has been challenged by Boissieras *et al.* who reported that iMab recognized C-rich sequences independently of iM-folding and that it might induce iM unfolding (17).

Prompted by the conflicting evidence, we set out to thoroughly investigate the selectivity of iMab using pull-down assays. We found that iMab binding is strongly influenced by experimental conditions and that the presence of the appropriate blocking agents and optimized salt concentrations are critical to avoid non-specific interactions. The use of skim milk and NaCl greatly improved the specific binding to iM forming sequences compared to unstructured DNA (Figure 2B–D). However, it should be noted that the optimized conditions reported here for the pull-down/WB experimental approach may not be readily applicable to other techniques, and any new technique, such as immunofluorescence or immunoprecipitation, requires proper setup and critical analysis of the results. For example, Schneekloth *et al.* developed iMab-based microarrays, in which different pH levels and crowding conditions were shown to cause slight changes in the iMab binding pattern; however, non-iM-forming sequences did not give a measurable response, regardless of the settings, thus confirming iMab specificity under these specific conditions (12). Ma *et al.* have shown that iM-folded synthetic oligonucleotides and sonicated chromatin from cells provided similar binding in a iMab-dot blot assay, while negative controls showed no signals, confirming iMab selectivity under these specific experimental conditions (9). iMab selectivity was further supported by whole genome sequencing approaches, where iMab-immunoprecipitated sequences were significantly enriched when compared to the negative control, both in a fragmented genomic DNA context (9,14) and in unfixed chromatin (11). In unfixed chromatin, the results obtained with iMab were strikingly similar to those obtained with BG4 for G4s (11,31,32), with the main difference being the level of expression of the genes presenting folded iMs versus G4s in their promoter. If iMab had recognized all C-rich sequences, this would not have been the case. In addition, iMab peaks were found to be enriched in iM forming sequences that were shown to fold *in vitro* at acidic pH. This last aspect further emphasizes that different folding conditions are expected *in vitro* and in cells.

Also, different *in vitro* techniques show different sensibility. For instance, while CD reports the average spectrum of all the conformations present in solution (Supplementary Figure S11), the pull-down/WB approach, by selecting only the antibody-bound conformation and then relying on signal amplification through HRP-conjugated antibodies, could detect even a small number of iM-folded molecules (Supplementary Figure S14B) (33).

An additional point of interest arising from our present results is the concentration of iMab and its target oligonucleotide, and the time of incubation necessary for iMab to recognize the target in the pull-down assay. iMab binds its iM target with high (nanomolar) affinity (7) and consequently retains binding strength towards iMs even when strongly diluted (Figure 2E). It may therefore be advisable to use as little amount of iMab as possible for each experiment to avoid unnecessary saturation and to increase selectivity. Similarly, iMab binding to the target occurs within the first 5 min of incubation and retains potency and selectivity as at longer incubation times (e.g. 1h). Therefore, shorter incubation times may be considered to improve the overall experimental procedure. Also, it should be considered that high DNA concentrations can stimulate the formation of intermolecular iM structures (26,27), as shown by NMR with the hTeloC variants used by Boissieras *et al.* and in this study (Figure 3A and B); thus the use of low DNA concentrations is advisable. To note that in NMR melting experiments the average properties of all the different structures present in solution are observed. As a consequence, (i) we cannot determine if the different structural elements (T-T, C^+^-C and A-T) belong to the same molecular species and if their contributions can be taken into account simultaneously, (ii) for the least stable species, the underdetermined baseline at low temperature reduces the accuracy of the *T*_1/2(NMR)_, (iii) the observed signal intensity of exchangeable protons is influenced not only by the unfolding of the structure but also by the rate of exchange with the solvent, and (iv) the width of the signals indicates that not only several intramolecular but also intermolecular species are present in solution. Even if the calculated *T*_1/2(NMR)_ in these conditions do not represent absolute values, the comparison of NMR spectra at various temperatures allows for the determination of the overall stability of the ensemble of structures present in solution (Supplementary Figures S5 and S6). Notably, the presence of flanking TAA residues, as used in the reference paper, appears to reduce but not completely inhibit the formation of intermolecular iMs *in vitro*. Indeed, the removal of these flanking nucleotides provided clearer results, confirming the formation of intermolecular iMs both in NMR and CD experiments. This finding offers a direct explanation for the apparent discrepancies with previous reported data (17) and validates the specificity of the iMab antibody.

Another critical point that recently raised concerns is iMab potential to unfold iMs (17). The possibility of quadruplex structure alteration upon binding with antibodies or proteins is a common consideration in such interaction studies. Previous research has demonstrated that the same protein can have opposing effects on quadruplex folding, depending on the target sequence. For instance, the human ribonucleoprotein K was found to bind iM structures with different outcomes: unfolding the *c-MYC* promoter sequence (34), while inducing iM folding in the HIV-1 LTR promoter (22). These findings suggest that multiple factors may influence the ultimate effect. The risk of structural changes is equally present with quadruplex-targeting antibodies. To date, we lack direct evidence of the actual folding state of quadruplexes in complex with antibodies, both for iMs/iMab and G4s/BG4, the most widely used anti-G4 antibody (21). Consequently, we primarily rely on biophysical assays performed on oligonucleotides. Evidence suggesting iM unfolding by iMab comes from a bulk-FRET assay, where Fluorescence resonance energy transfer (FRET) efficiency decreases upon iMab binding (17). While this result could indicate structure unfolding, as proposed by the authors, it might also imply a rearrangement of the iM structure, with the antibody increasing the distance between terminal fluorophores while preserving the intercalated structure. To address this issue, we conducted CD analysis on sequences with intermediate iM stability in different folding conditions in the absence and presence of iMab. Our results demonstrate that the oligonucleotide iM conformation, whether it is folded or unfolded, remains primarily unaffected by the antibody (Supplementary Figure S13). These findings support the hypothesis that in the bulk-FRET experiment, iMab positions itself between the two fluorophores, increasing their distance and thus reducing FRET efficiency, rather than unfolding the structure.

Overall, our results provide new insights into the iM field and demonstrate that iMab can be used to detect intermolecular iMs *in vitro*. With the presence of intermolecular G4s in cells recently reported to be involved in long-range distance interactions (35), this raises the intriguing possibility that such intermolecular structures could also be formed by iMs *in vivo*, and that such a scenario could be confirmed by iMab binding. Indeed, iMs were found to be particularly enriched in gene promoters (11), which are known to be engaged in long distance interaction with distal elements to regulate chromatin 3D organization (36). Also, iMs were found to be located at the centromere level, where the formation of interchromosomal interactions is well established and reported to regulate gene transcription (37). Formation of intermolecular quadruplexes, both iMs and G4s, might mediate these processes, contributing to the fundamental gene-regulatory networks.

## Supplementary Material

gkae1305_Supplemental_File

## Data Availability

The data underlying this article are available in the article and in its online supplementary material.
