# Supplementary material for: The iMab antibody selectively binds to intramolecular and intermolecular i-motif structures
Source: Nucleic Acids Res. 2025 Jan 15;53(2):gkae1305. doi: 10.1093/nar/gkae1305 (PMC11734697; doi:10.1093/nar/gkae1305)
Supplement: gkae1305_Supplemental_File [file gkae1305_supplemental_file.pdf]

Supporting information

The iMab antibody selectively binds to intramolecular and intermolecular i-motif structures

Emanuela Ruggiero[1], Maja Marušič[2], Irene Zanin[1], Cristian David Peña Martinez[3,4], Daniel Christ[3,4#], Janez Plavec[2#] and Sara N. Richter[1,5#*]

[1]Department of Molecular Medicine, University of Padua, 35121 Padua, Italy

[2]Slovenian NMR Centre, National Institute of Chemistry, Hajdrihova 19, SI-1000 Ljubljana, Slovenia

[3]Garvan Institute of Medical Research, Darlinghurst, Sydney NSW 2010, Australia

[4]St Vincent's Clinical School, Faculty of Medicine, University of New South Wales, Kensington, Sydney NSW 2010, Australia

[5]Microbiology and Virology Unit, Padua University Hospital, 35121 Padua, Italy

[#] Senior authors

*To whom correspondence should be addressed: sara.richter@unipd.it

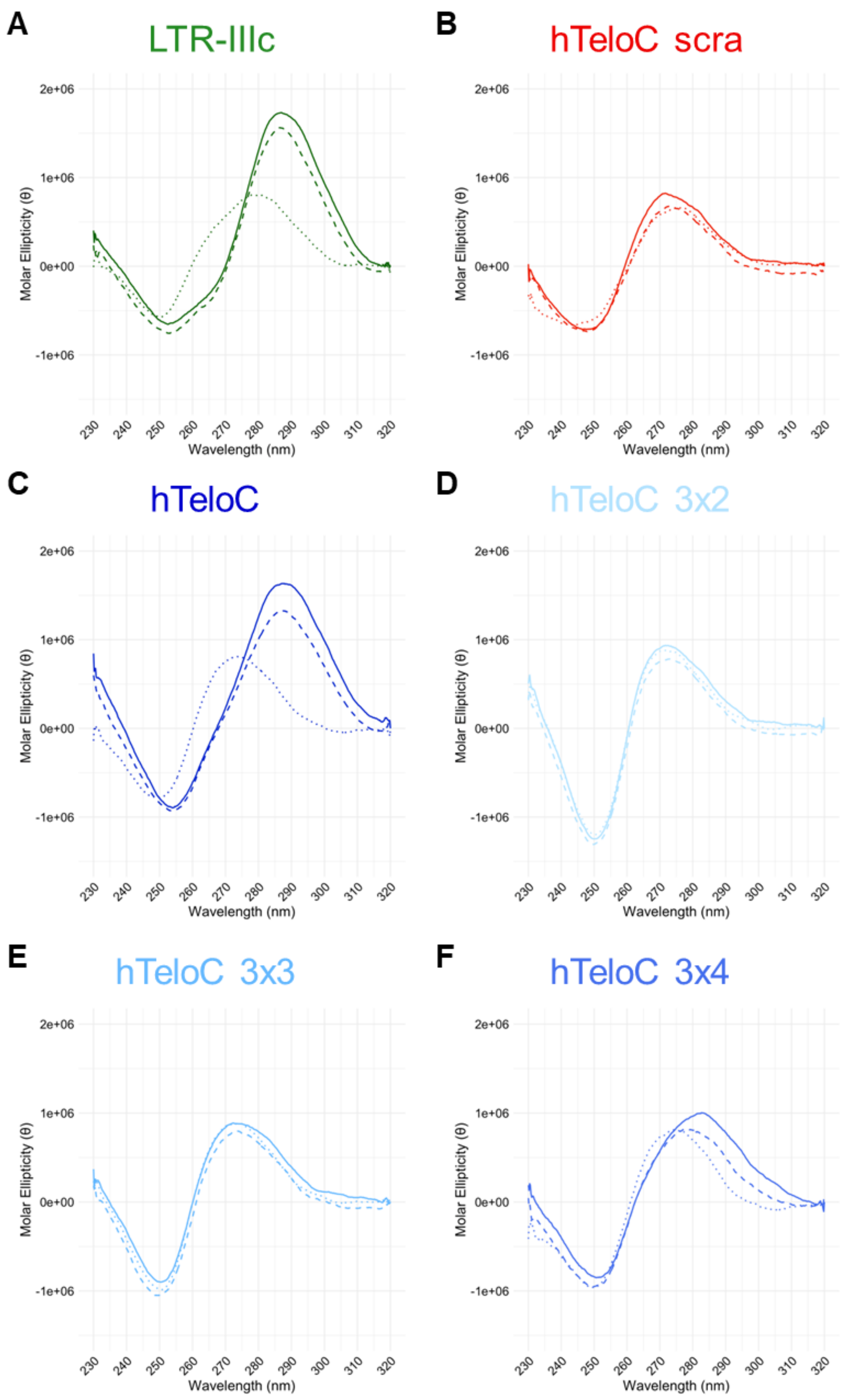

Figure S1. CD spectra of selected unmodified sequences. Samples were prepared in 20 mM phosphate buffer at pH 5.4 (plain line), pH 6.0 (dashed line) and pH 7.4 (dotted line), with 80 mM KCl at 3 µM final concentration. Molar ellipticity (θ) was measured at 20°C and reported as θ = deg x cm^2 x $dmol^{-1}$.

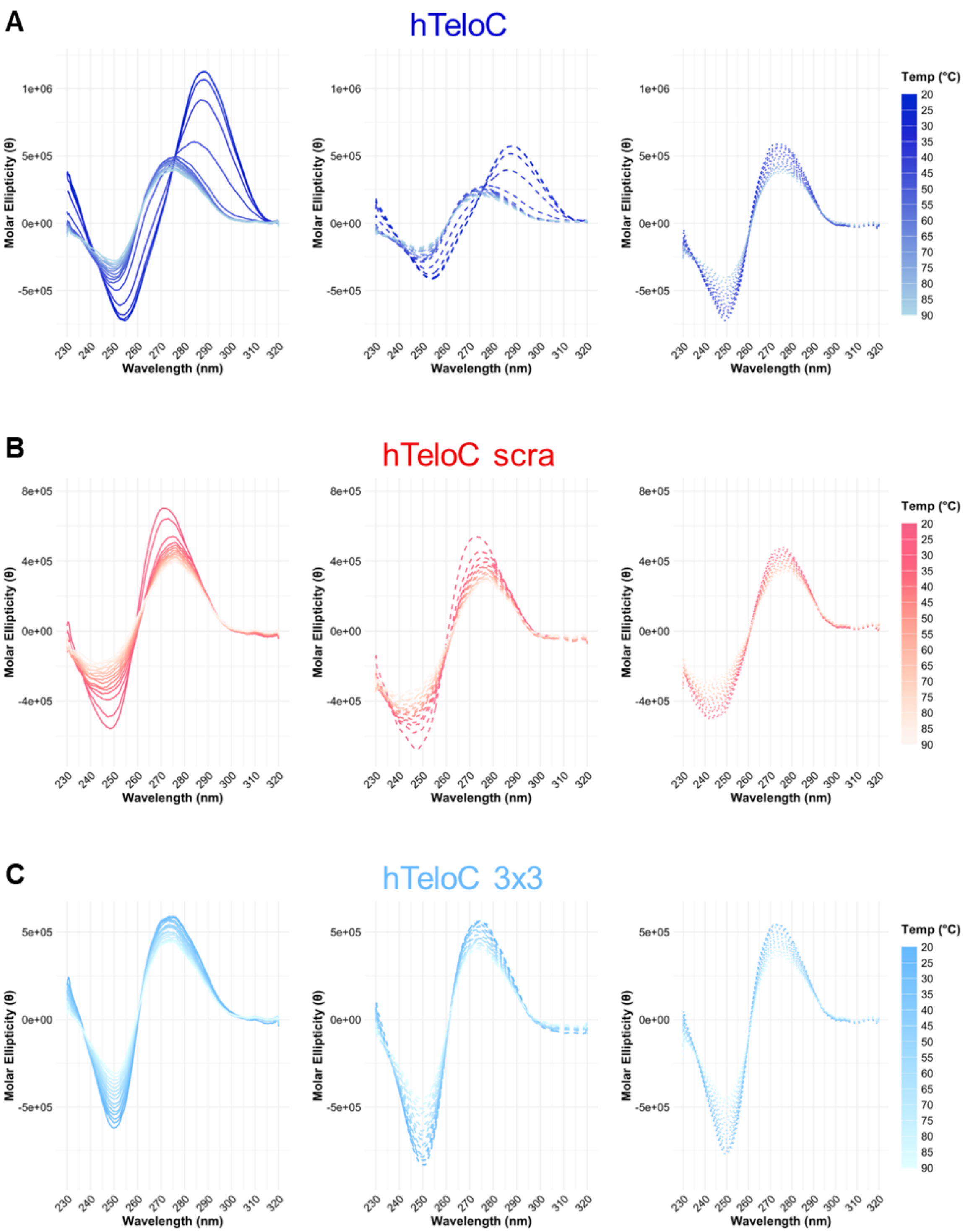

Figure S2. CD thermal unfolding spectra of biotinylated selected sequences. Samples were prepared in 20 mM phosphate buffer at pH 5.4 (plain lines, left panel), pH 6.0 (dashed lines, center panel) and pH 7.4 (dotted lines, right panel), with 80 mM KCl at 3 µM final concentration. Molar ellipticity (θ) was measured over a temperature range of 20-90°C and reported as $\theta = \text{deg} \times \text{cm}^2 \times \text{dmol}^{-1}$.

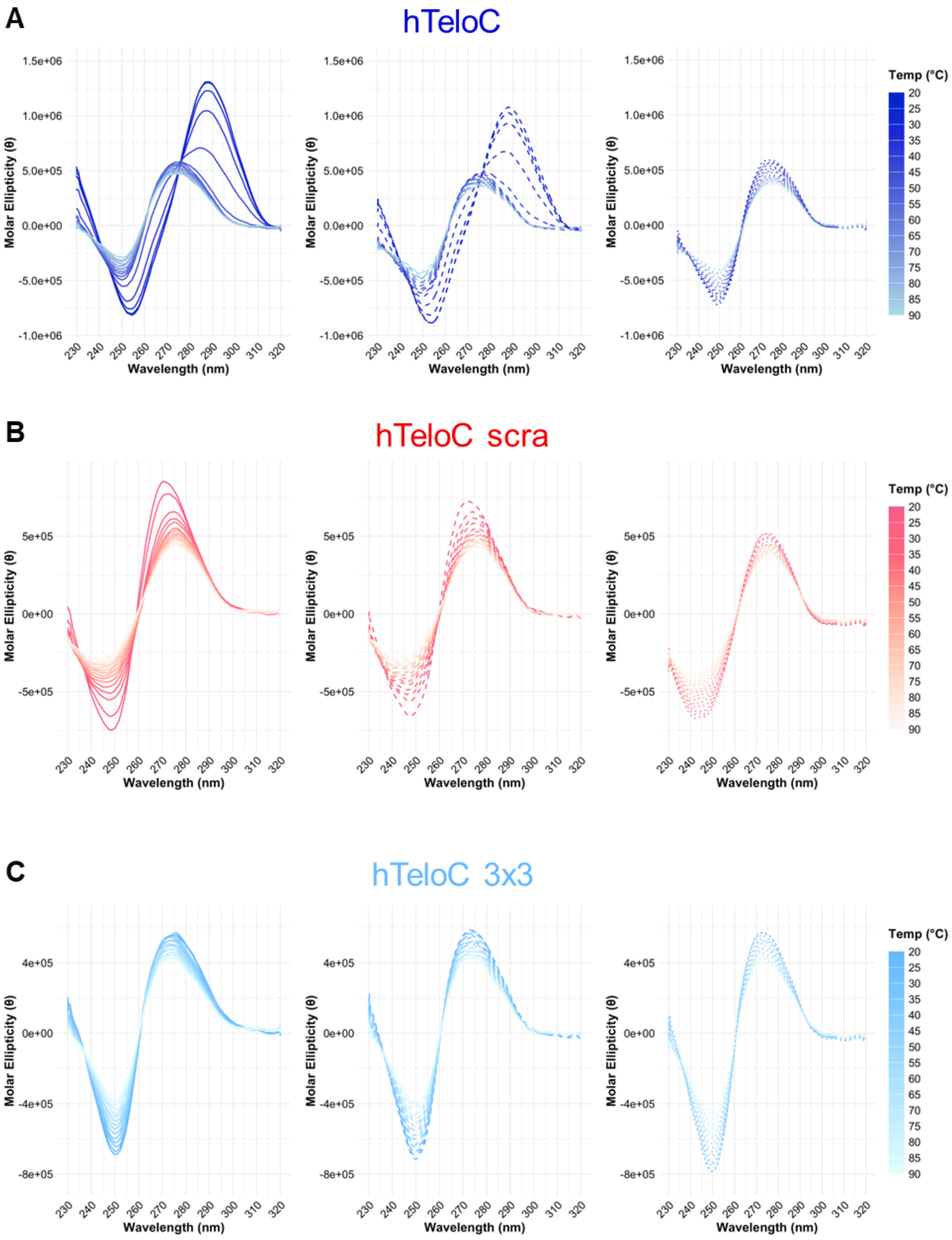

Figure S3. CD thermal unfolding spectra of unmodified selected sequences. Samples were prepared in 20 mM phosphate buffer at pH 5.4 (plain lines, left panel), pH 6.0 (dashed lines, center panel) and pH 7.4 (dotted lines, right panel), with 80 mM KCl at 3 μM final concentration. Molar ellipticity (θ) was measured over a temperature range of 20-90°C and reported as θ = deg × cm^2 × $dmol^{-1}$.

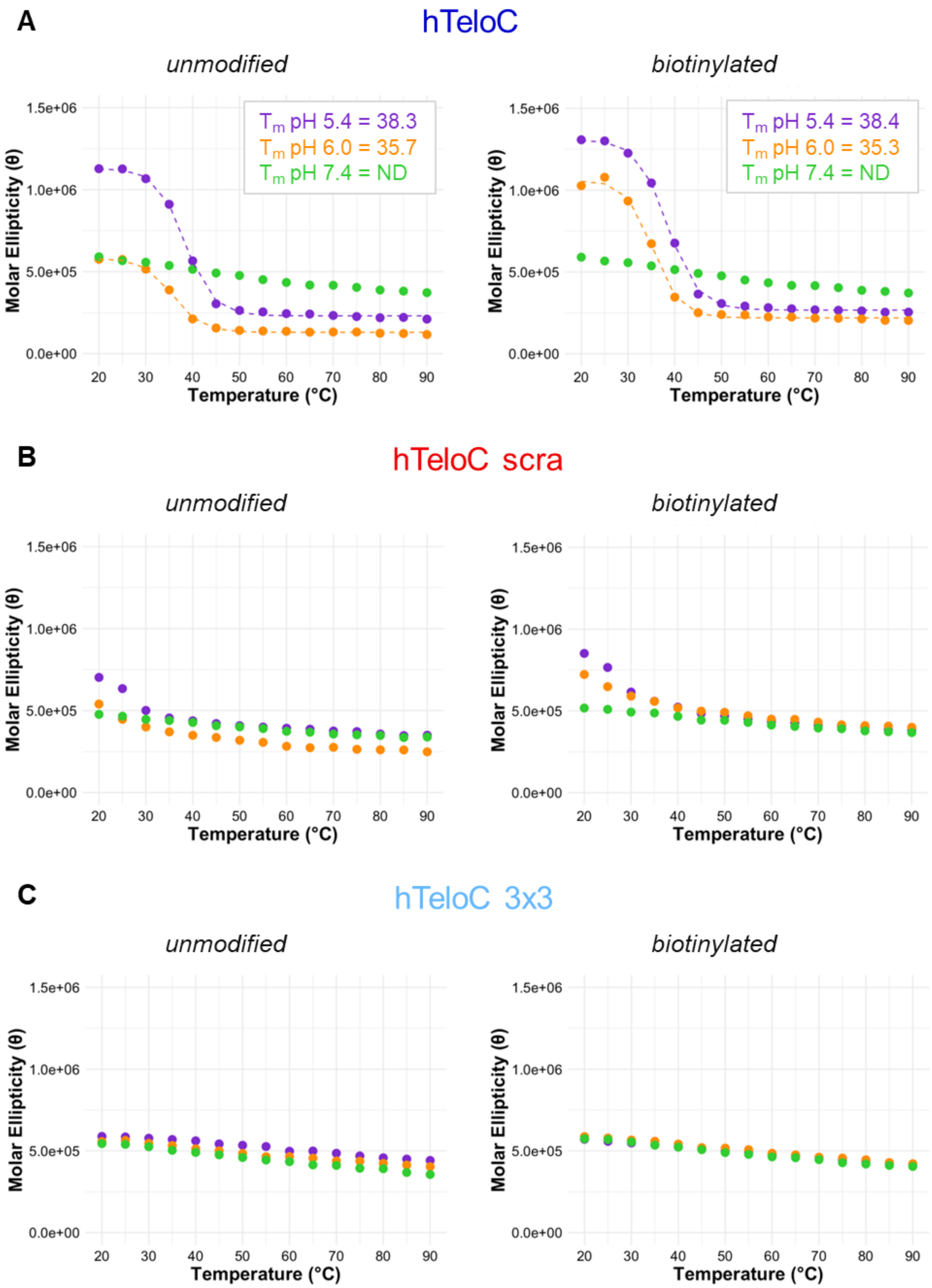

Figure S4. CD melting curves of selected sequences. Samples were prepared in 20 mM phosphate buffer at pH 5.4 (purple), pH 6.0 (orange) and pH 7.4 (green), with 80 mM KCl at 3 μM final concentration. Molar ellipticity (θ) was measured over a temperature range of 20-90°C and reported as θ = deg × cm^2 × dmol^{-1}. Data are reported as the molar ellipticity at peak wavelength as a function of the temperature.

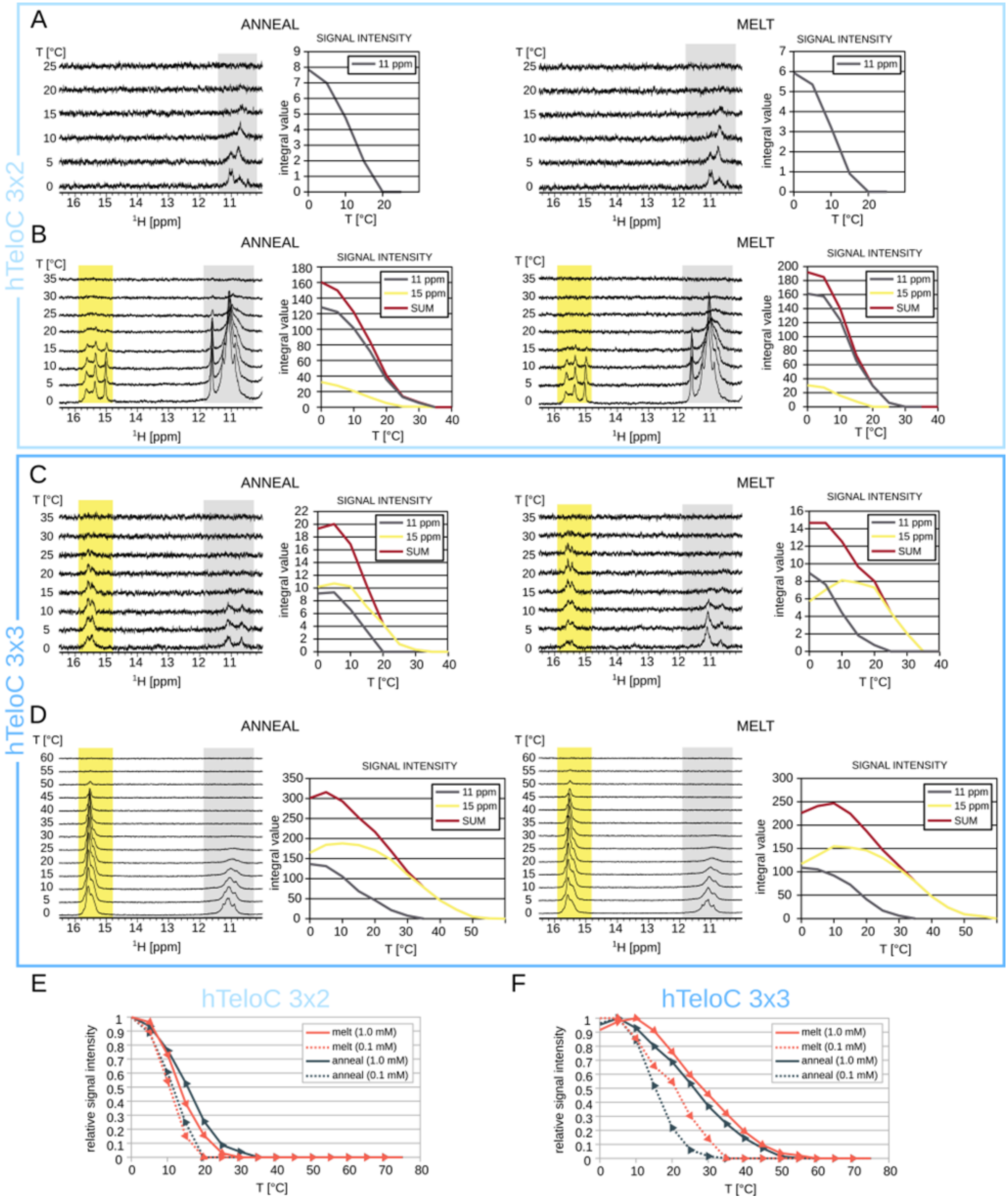

Figure S5: Analysis of NMR melt and anneal experiments and contribution of the base-pair type to the overall signal intensity. Imino regions of 1D 1H NMR spectra of A) hTeloC 3x2 at 0.1 mM, B) hTeloC 3x2 at 1 mM, C) hTeloC 3x3 at 0.1 mM and D) hTeloC 3x3 at 1 mM concentration together with analysis of signal intensity in C-C+, T-T and joint imino region at different temperatures. C-C+, and T-T regions are marked with yellow and gray rectangles, respectively. Melt (red) and anneal (dark gray) curves for E) hTeloC 3x2 and F) hTeloC 3x3 at different oligonucleotide concentrations based on the overall intensity of the signals in the imino region of 1D proton NMR spectra. Spectra were recorded in buffer with 80 mM KCl and 20 mM potassium phosphate buffer at pH 5.4.

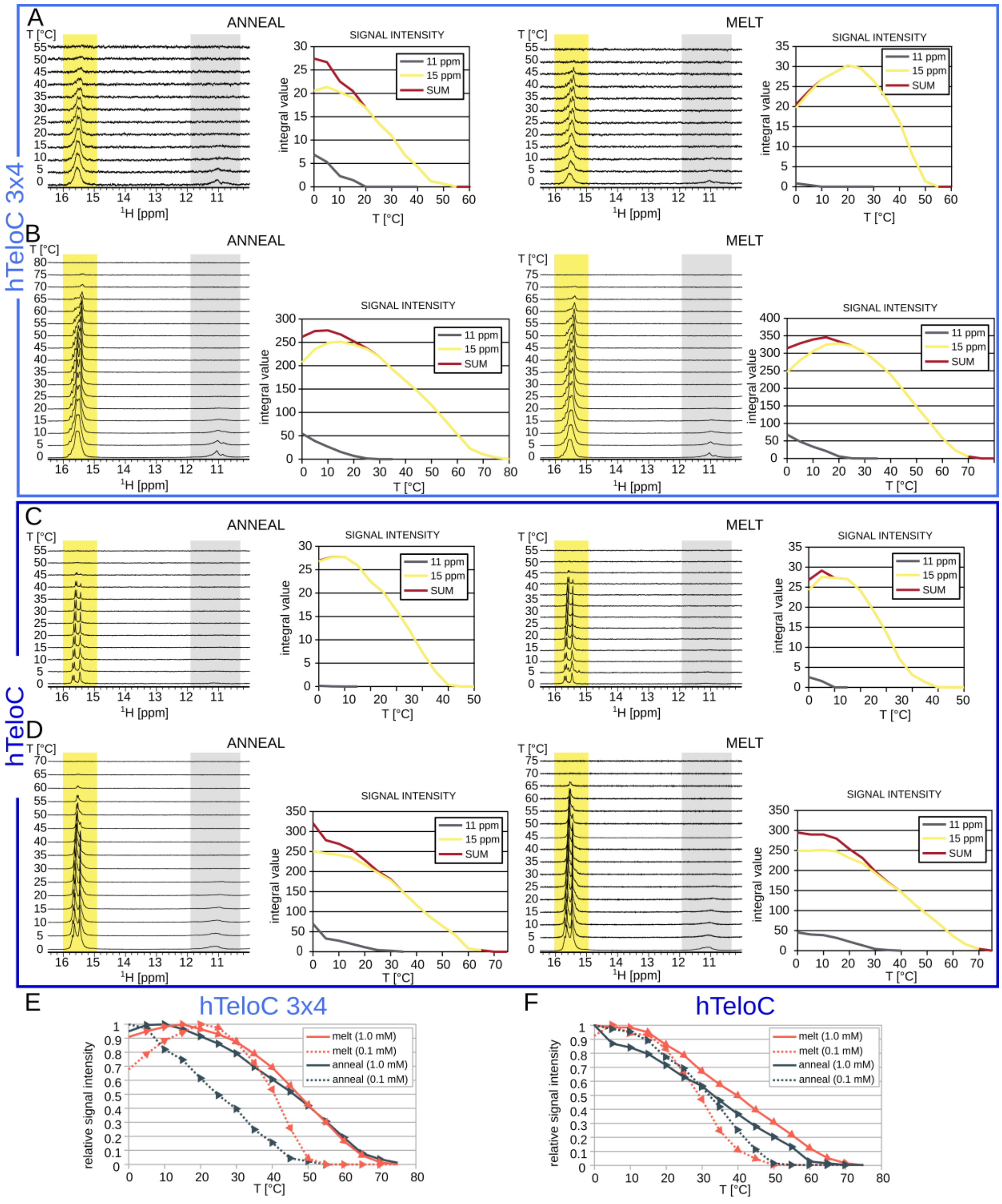

Figure S6: Analysis of NMR melt and anneal experiments and contribution of the base-pair type to the overall signal intensity. Imino regions of 1D ^{1}H NMR spectra of A) hTeloC 3x4 at 0.1 mM, B) hTeloC 3x4 at 1 mM, C) hTeloC at 0.1 mM and D) hTeloC at 1 mM concentration together with analysis of signal intensity in C-C$^+$, T-T and joint imino region at different temperatures. C-C$^+$, and T-T regions are marked with yellow and gray rectangles, respectively. Melt (red) and anneal (dark gray) curves for E) hTeloC 3x4 and F) hTeloC at different oligonucleotide concentrations based on the overall intensity of the signals in the imino region of 1D proton NMR spectra. Spectra were recorded in buffer with 80 mM KCl and 20 mM potassium phosphate buffer at pH 5.4.

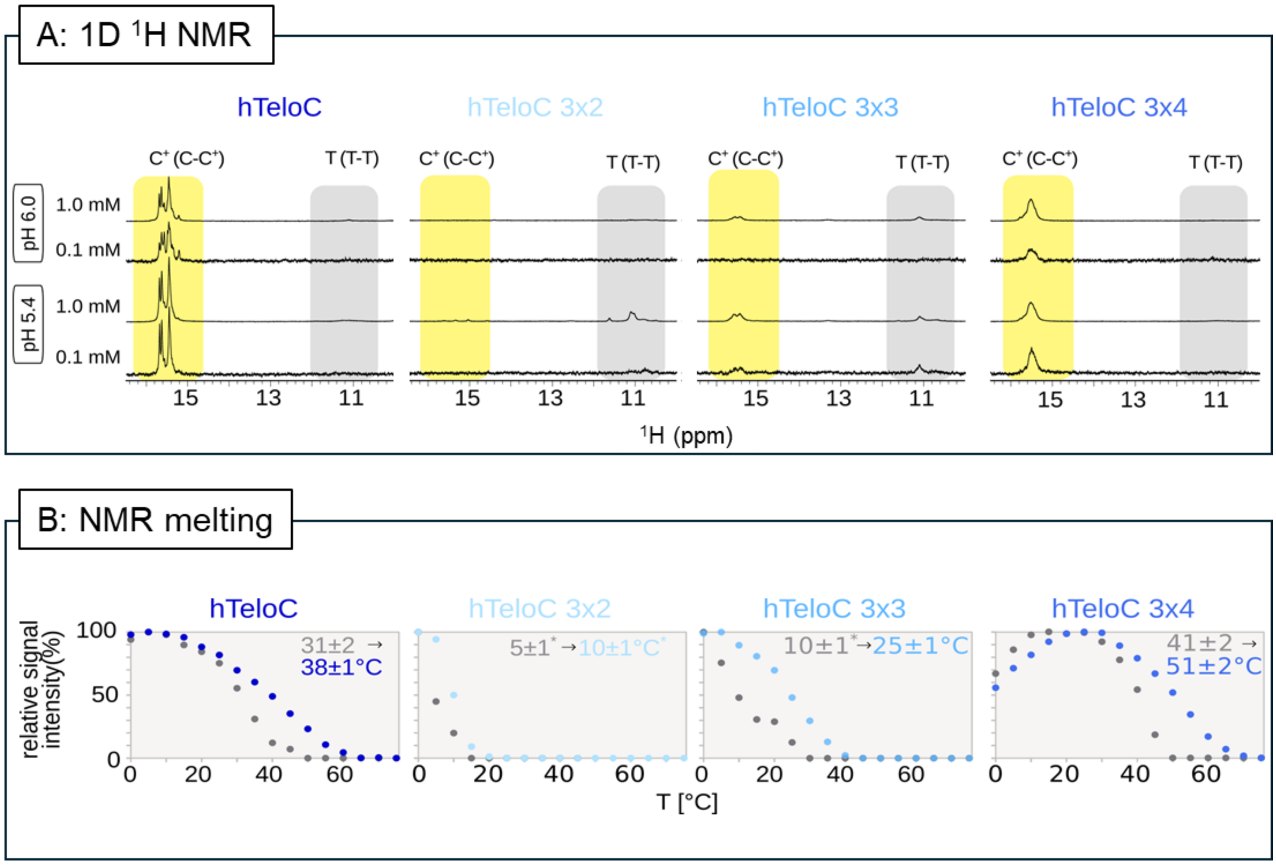

Figure S7. iMab binding profile to multimolecular iMs. A) Imino region of 1D ^{1}H NMR spectra recorded at 5°C, at different pH and at oligonucleotide concentrations, in the presence of $MgCl_2$. Regions characteristic for signals of protons included in non-canonical C-C^{+} and T-T base-pairs are designated with yellow and grey highlighted areas, respectively. Vertical scale of spectra at 1.0 mM oligonucleotide concentration is reduced by a factor of 10. B) $T_{1/2}$(NMR) at two different oligonucleotide concentrations obtained from NMR melt experiments based on the intensity of the signals in the imino region. Data points in the melting profiles displayed in gray represent data at 0.1 mM oligonucleotide concentration, while colored data points represent data at 1.0 mM oligonucleotide concentration.

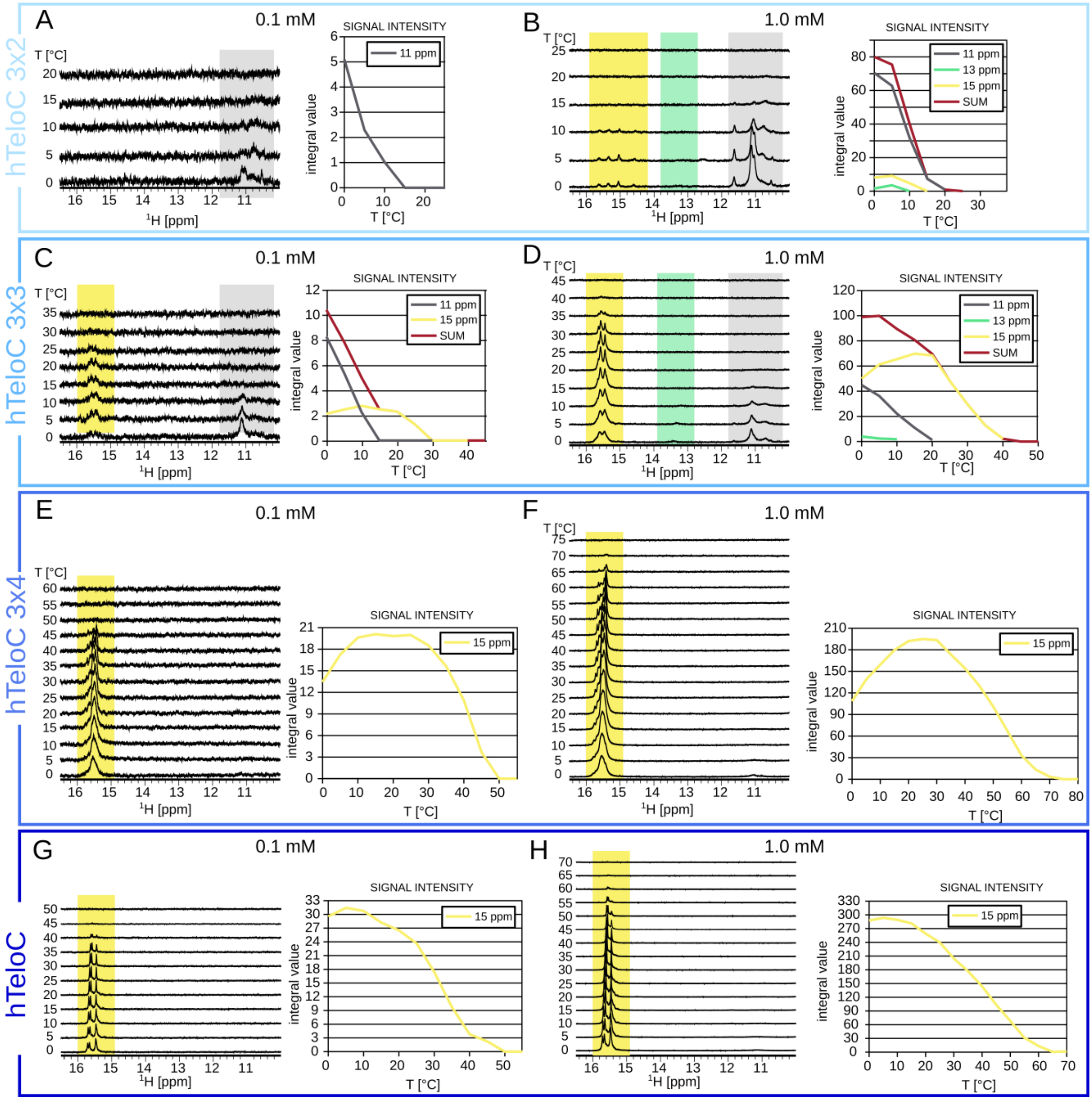

Figure S8: Analysis of NMR melting experiments and contribution of the base-pair type to the overall signal intensity, measured in the presence of MgCl2. Imino regions of 1D 1H NMR spectra of A) hTeloC 3x2 at 0.1 mM, B) hTeloC 3x2 at 1.0 mM, C) hTeloC 3x3 at 0.1 mM, D) hTeloC 3x3 at 1.0 mM, E) hTeloC 3x4 at 0.1 mM, F) hTeloC 3x4 at 1.0 mM, G) hTeloC at 0.1 mM and H) hTeloC at 1.0 mM concentration together with analysis of signal intensity in C-C+, T-T and joint imino region at different temperatures. C-C+, A-T and T-T regions are marked with yellow, green and gray rectangles, respectively. Spectra were recorded in buffer with 1 mM $MgCl_2$, 10 mM KCl and 100 mM potassium phosphate buffer at pH 5.4.

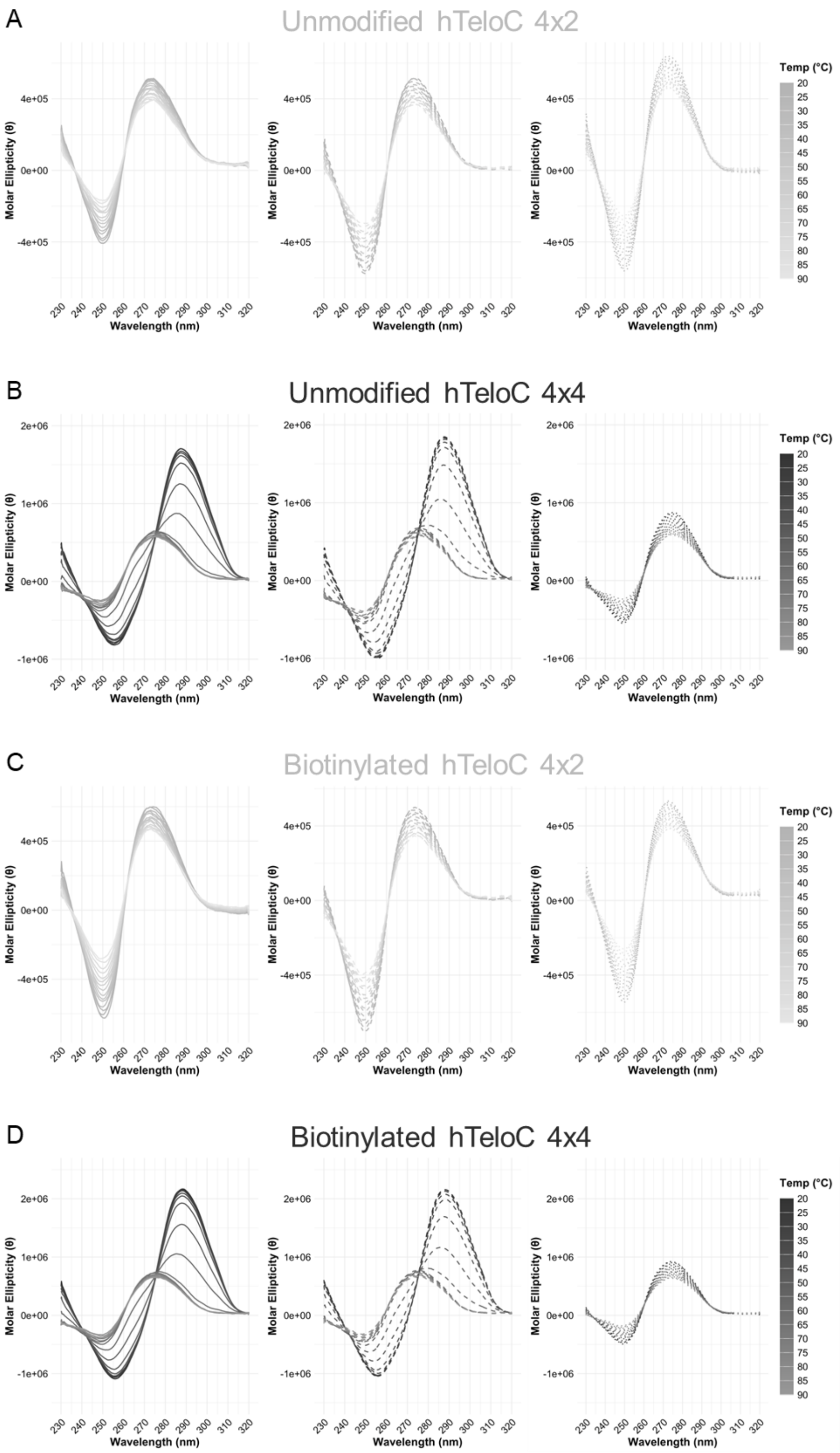

Figure S9. CD thermal unfolding of 4 C-tract sequences, hTeloC 4x2 (A-B) and 4x4 (C-D). Samples were prepared in 20 mM phosphate buffer at pH 5.4 (plain lines, left panel), pH 6.0 (dashed lines, center panel) and pH 7.4 (dotted lines, right panel), with 80 mM KCl at 3 µM final concentration. Molar ellipticity (θ) was measured over a temperature range of 20-90°C and reported as θ = deg × cm^2 × $dmol^{-1}$. Data are reported as the molar ellipticity at peak wavelength as a function of the temperature

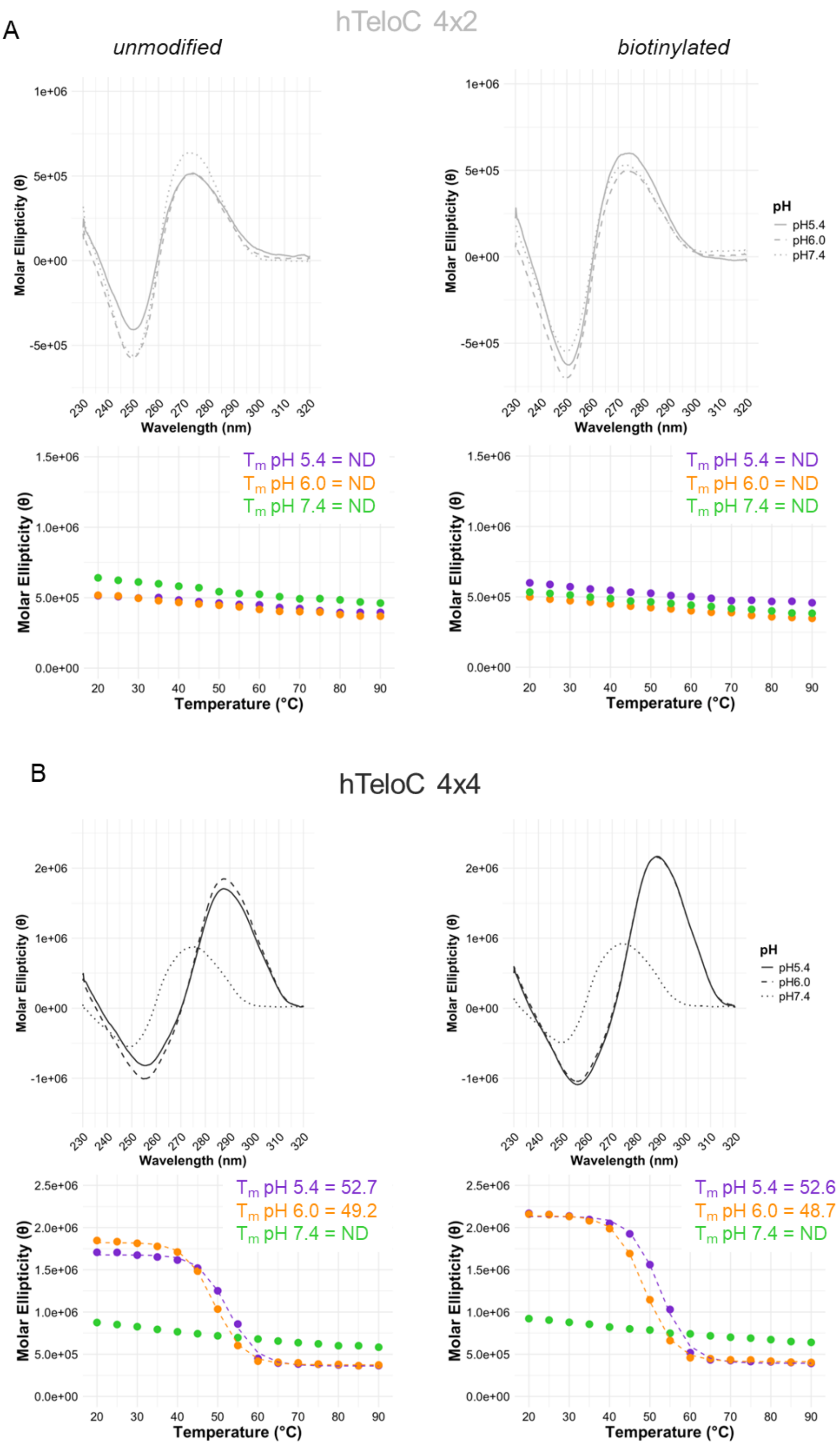

Figure S10. CD analysis of hTeloC 4x2 (A) and hTeloC 4x4 (B). The analysis was performed on both unmodified (left panels) and biotinylated (right panels) oligonucleotides. For each sequence, CD spectra measured at 20 and melting curves measured pH 5.4 (plain lines), pH 6.0 (dashed lines) and pH 7.4 (dotted lines) are reported. Molar ellipticity (θ) was measured at 20°C and reported as θ = deg x cm^2 x dmol^{-1}.

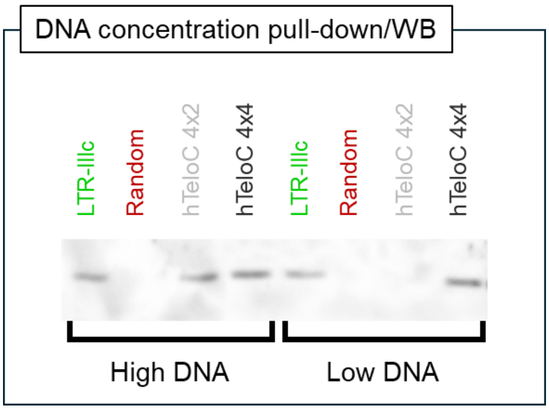

Figure S11. Pull-down/WB performed at low (300 nM) and high (1500 nM) DNA amounts, with 10 ng iMab per sample.

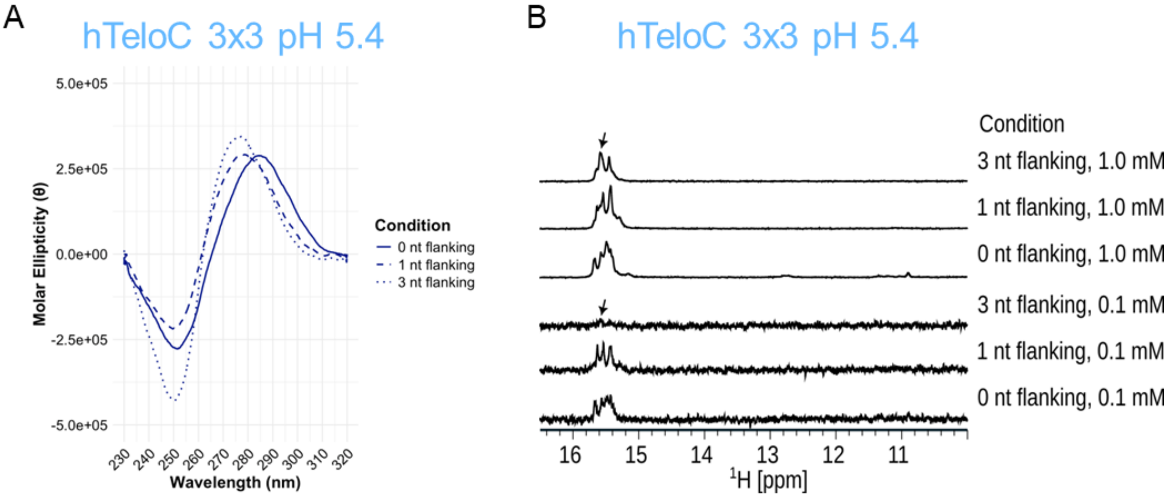

Figure S12. Effect of the flanking nucleotides on iM folding. A) CD spectra of the hTeloC 3x3 sequence in the absence of flanking nucleotides (plain line), 1 flanking nucleotide (dashed line) or three flanking nucleotides (dotted line). Samples were prepared in phosphate buffer at pH 5.4; molar ellipticity (θ) was measured at 20°C and reported as θ = deg x cm^2 x $dmol^{-1}$. B) ^{1}H NMR spectra of selected sequences at pH 5.4, 25°C and two different concentrations. The arrow indicates a characteristic signal of hTeloC 3x3 3 nt flanking at 1 mM concentration that can be recognized in the imino region of hTeloC 3x3 3 nt flanking at 0.1 mM concentration. Vertical scale of spectra at 1.0 mM oligonucleotide concentration is reduced by a factor of 10.

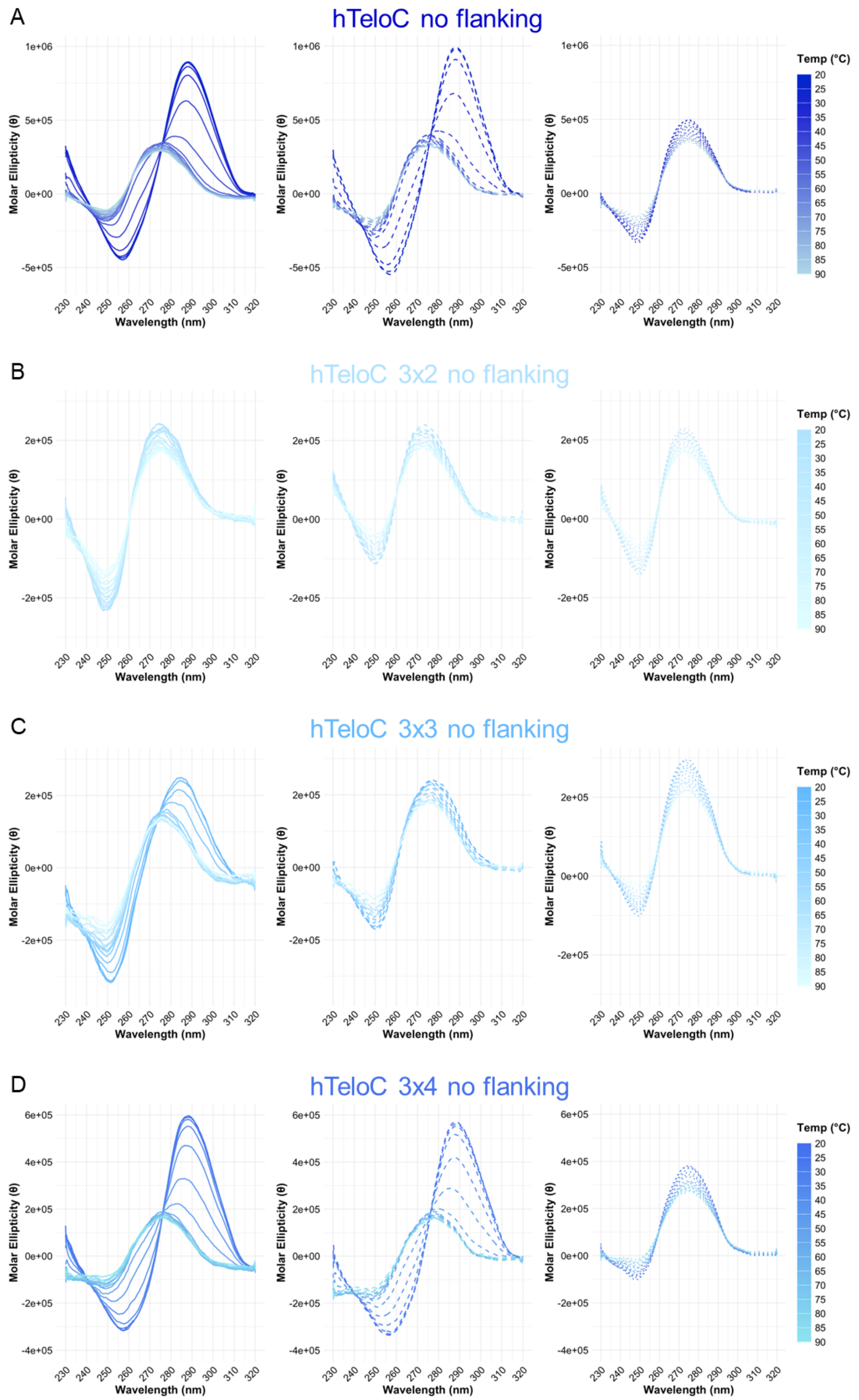

Figure S13. CD thermal unfolding spectra of the hTeloC no flanking (nf)-derived sequences in the absence of the TAA flanking regions. Samples were prepared in phosphate buffer at pH 5.4 (plain line), pH 6.0 (dashed line) and pH 7.4 (dotted line), at a final concentration of 3 μM. Molar ellipticity (θ) was measured over a temperature range of 20-90°C and reported as θ = deg x cm^2 x $dmol^{-1}$.

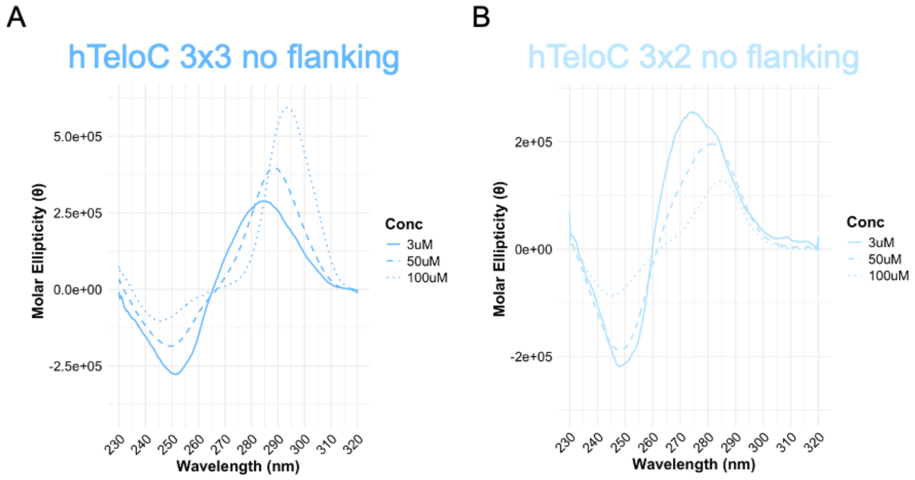

Figure S14. Effect of the oligonucleotides concentration on iM folding. CD spectra of the hTeloC 3x3nf (A) and hTeloC 3x2nf (B) sequences at 3 μM (plain line), 50 μM (dashed line) or 100 μM (dotted line). Samples were prepared in phosphate buffer at pH 5.4; molar ellipticity (θ) was measured at 20°C and reported as θ = deg x cm^2 x dmol^{-1}.

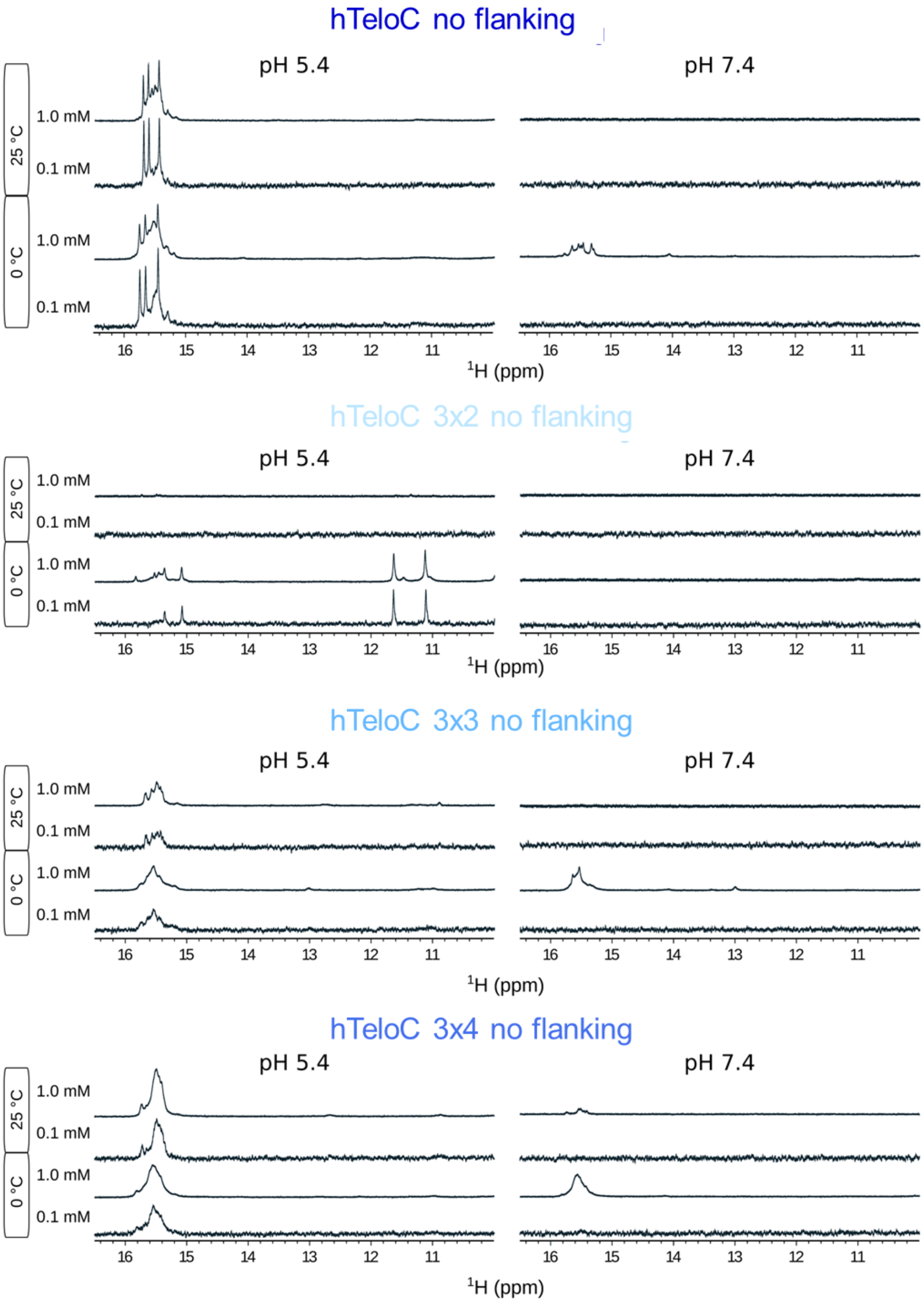

Figure S15. Effect of temperature, pH and oligonucleotide concentration on folding and stability of hTeloC-derived sequences in the absence of the TAA flanking regions. Vertical scale of spectra at 1.0 mM oligonucleotide concentration is reduced by a factor of 10. NMR spectra were recorded at 0 or 25°C, acidic or neutral pH, and 0.1 or 1.0 mM oligonucleotide concentration in the presence of 20 mM potassium phosphate buffer of indicated pH and 80 mM KCl.

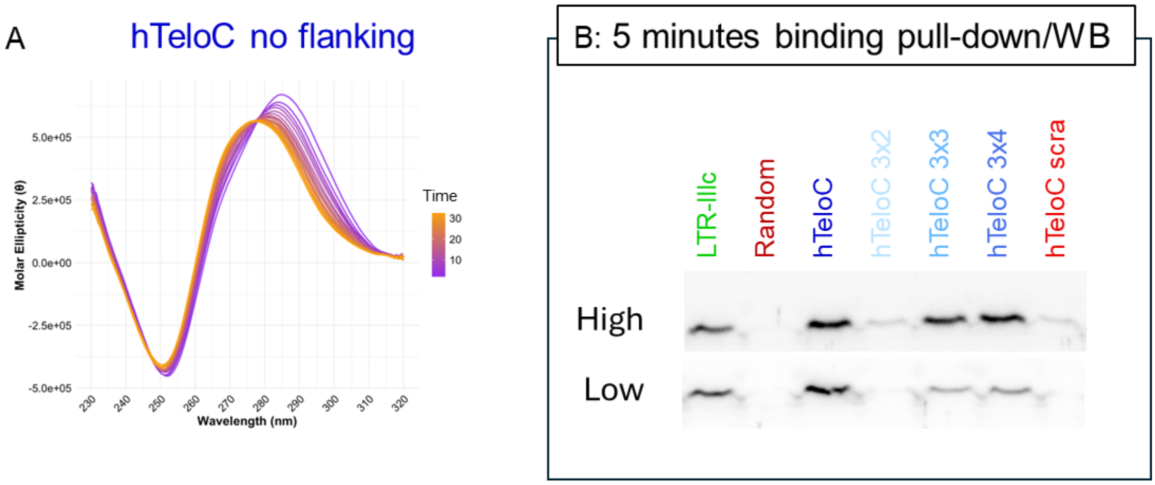

Figure S16. A) CD spectra of the hTeloC sequence in the presence of K_2HPO_4, recorded every two minutes. Samples were prepared in phosphate buffer at pH 6.0, denatured and cooled overnight. Then, phosphate buffer was added up to pH 7.4 and the spectra were immediately recorded. Molar ellipticity (θ) was measured at 20°C for each spectrum and reported as $\theta = deg \times cm^2 \times dmol^{-1}$. B) Pull-down/WB performed at low (300 nM) and high (1500 nM) DNA amounts, with 10 ng iMab per sample, incubated for 5 minutes.

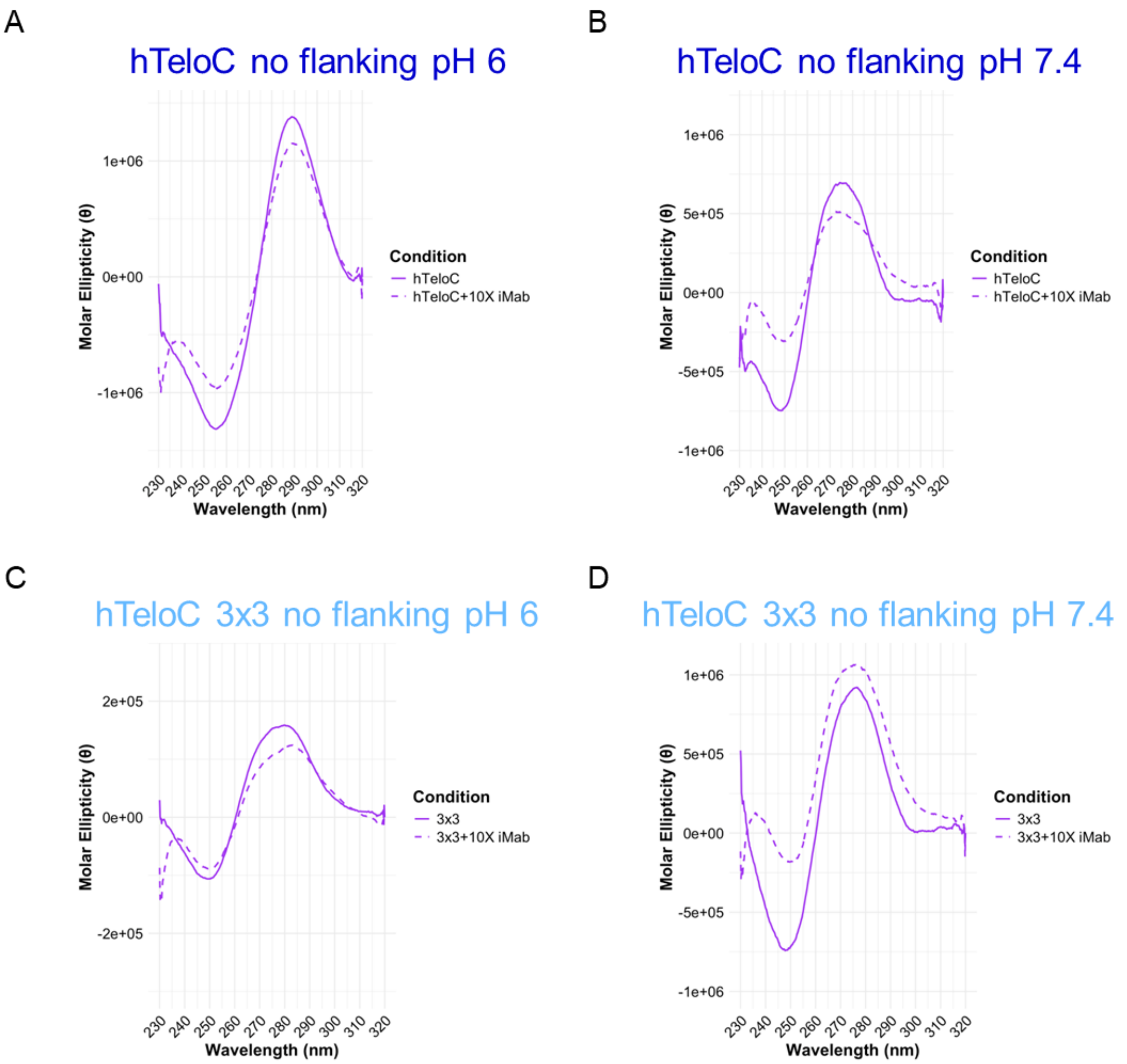

Figure S17. Effect of iMab on iM conformation. CD spectra of hTeloC and hTeloC 3x3 sequences performed in the absence (A, C) and presence (B, D) of ten-fold molar excess of iMab. Samples were prepared in phosphate buffer at indicated pH, at a final concentration of 1.5 μM; molar ellipticity (θ) was measured at 20°C and reported as θ = deg x cm^2 x $dmol^{-1}$.
